# Effectiveness of aerobic exercise for adults living with HIV: systematic review and meta-analysis using the Cochrane Collaboration protocol

**DOI:** 10.1186/s12879-016-1478-2

**Published:** 2016-04-26

**Authors:** Kelly K. O’Brien, Anne-Marie Tynan, Stephanie A. Nixon, Richard H. Glazier

**Affiliations:** Department of Physical Therapy, University of Toronto, 500 University Avenue, Room 160, Toronto, ON Canada; Rehabilitation Sciences Institute (RSI), University of Toronto, 500 University Avenue, Room 160, Toronto, ON Canada; Institute of Health Policy, Management and Evaluation (IHPME), University of Toronto, Toronto, ON Canada; Centre for Research on Inner City Health, Li Ka Shing Knowledge Institute, St. Michael’s Hospital, Toronto, ON Canada; Institute for Clinical Evaluative Sciences, G1 06 2075 Bayview Ave, Toronto, ON Canada; Department of Family and Community Medicine, St. Michael’s Hospital, 30 Bond Street, Toronto, ON Canada; Department of Family and Community Medicine, University of Toronto, 500 University Avenue, Toronto, ON Canada

**Keywords:** HIV/AIDS, Exercise, Aerobic, Systematic review

## Abstract

**Background:**

People with HIV are living longer with the health-related consequences of HIV, multi-morbidity, and aging. Exercise is a key strategy that may improve or sustain health for people living with HIV. Our aim was to examine the safety and effectiveness of aerobic exercise interventions on immunological, virological, cardiorespiratory, strength, weight, body composition, and psychological outcomes in adults living with HIV.

**Methods:**

We conducted a systematic review using the Cochrane Collaboration protocol. We searched databases up to April 2013. We included randomized controlled trials comparing aerobic exercise with no exercise or another intervention performed at least three times per week for at least four weeks among adults living with HIV. Two reviewers independently determined study eligibility. Data were extracted from studies that met inclusion criteria using standardized forms. We assessed risk of bias using the Cochrane Collaboration’s tool for assessing risk of bias. Outcomes were analyzed as continuous and meta-analyses conducted using random effects models with Review Manager (RevMan) computer software.

**Results:**

Twenty-four studies met inclusion criteria (*n* = 936 participants at study completion); the majority of participants were men (73 %) and the majority were taking antiretroviral therapy (19/24 included studies). The exercise intervention included aerobic exercise alone (11 studies) or a combination of aerobic and resistive exercise (13 studies) ranging from 5 to 52 weeks. Fifty-eight meta-analyses were performed. Main results indicated statistically significant improvements in selected outcomes of cardiorespiratory status (maximum oxygen consumption, exercise time), strength (chest press, knee flexion), body composition (lean body mass, percent body fat, leg muscle area), depression symptoms, and quality of life (SF-36 questionnaire) among exercisers compared with non-exercisers. No significant differences in change in CD4 count and viral load were found.

**Conclusions:**

Performing aerobic exercise or a combination of aerobic and resistive exercise at least three times per week for at least five weeks is safe and can lead to improvements in cardiorespiratory fitness, strength, body composition and quality of life for adults with HIV. Aerobic exercise is safe and beneficial for adults living with HIV who are medically stable.

**Electronic supplementary material:**

The online version of this article (doi:10.1186/s12879-016-1478-2) contains supplementary material, which is available to authorized users.

## Background

Access to combination antiretroviral therapy has transformed HIV into a chronic illness whereby many individuals are living longer and aging with the health-related consequences of HIV, adverse effects of treatment and multi-morbidity [1–4]. These consequences may be known as “disability” including symptoms and impairments (problems with body function or structure, such as pain or fatigue), activity limitations (difficulties in executing day-to-day activities, such as inability to walk), challenges to social inclusion (problems in life situations, such as inability to work, personal relationships), and uncertainty about future health (worrying about the future) [5, 6].

Exercise is a strategy employed by people living with HIV and by rehabilitation professionals to address disability and improve or sustain the health of people living with HIV [7]. Exercise has been shown to improve strength, cardiovascular function, and psychological status in general populations [8, 9]. Similar benefits of aerobic exercise were documented in earlier versions of this systematic review among adults living with HIV [10, 11]. However, knowledge about the benefits and risks of exercise, and optimal parameters for exercise for adults living with HIV is still emerging. If the risks and benefits of exercise for people living with HIV are better understood, appropriate exercise may be undertaken by people in this population and appropriate exercise prescription may be practiced by healthcare providers. Effective and safe exercise may enhance the effectiveness of HIV management, thus improving overall health outcomes for adults living with HIV.

Our aim was to examine the safety and effectiveness of aerobic exercise interventions on immunological, virological, cardiorespiratory, strength, weight, body composition, and psychological outcomes in adults living with HIV.

## Methods

We conducted a systematic review using the Cochrane Collaboration protocol [12].

### Inclusion criteria

We included randomized controlled trials (RCTs) comparing aerobic exercise (or combined aerobic and resistance exercise) with no aerobic exercise or another exercise or treatment modality performed at least three times per week for at least four weeks [13]. We included studies of adults (18 years of age and older) living with HIV at all stages of infection with or without comorbidities. We defined aerobic exercise as a regimen containing aerobic interventions performed at least three times per week for at least four weeks. Aerobic interventions included but were not limited to walking, jogging, cycling, rowing, stair stepping, and swimming. Interventions may or may not have been supervised [13].

### Outcomes

We assessed immunological (CD4 count, cells/mm^3^) and virological (viral load, log10 copies) outcomes. Cardiorespiratory measures included but were not limited to maximal oxygen consumption (VO2max), exercise time, oxygen pulse, maximum heart rate, maximum tidal volume, minute ventilation, lactic acid threshold (LAT), maximum work rate, and rate of perceived exertion. Strength measures included amount of weight able to resist in kilograms (1-repetition maximum) for major muscle groups. Weight and body composition measures included any outcome that contributes to the direct or indirect measurement of muscle, fat, bone or other tissues of the body. These included but were not limited to body weight, body mass index (BMI), lean body mass, girth, percent body fat, cross-sectional muscle area, and waist and hip circumference. Psychological measures included general measures of psychological status and health-related quality of life.

### Search strategy

In the update of this systematic review, we searched databases from 2009 to April 2013 including Medline, Cochrane Central Register of Controlled Trials, Cochrane Database of Systematic Reviews, Database of Abstracts of Reviews of Effects, PsycINFO, CINAHL, EMBASE, Web of Science: Science Citation Index, SPORTdiscus, Virology and AIDS Abstracts and LILACS. We also searched clinicaltrials.gov and reference lists from pertinent articles. All languages were included. See Additional file 1 for the detailed MEDLINE search strategy which we modified as needed for use with other databases.

### Selection of included studies

All abstracts retrieved from the search were reviewed independently by two reviewers (KKO and AMT) who applied the following four inclusion criteria to determine if the abstract warranted further investigation: a) Did the study include human participants who were HIV positive? b) Did the study include adults 18 years of age or older? c) Did the study include an aerobic exercise intervention performed at least three times/week, at least 20 min per session for at least four weeks? d) Was there a randomized controlled comparison group?

When the review based on the abstract alone indicated that one or both raters believed the study met eligibility criteria (i.e., if reviewers answered “yes” or “unsure” to the four questions) then full versions of the article were independently reviewed by the two reviewers to determine article inclusion. In instances where there was a lack of agreement by the two reviewers, a third reviewer was asked to review the full article to determine final inclusion.

### Data extraction

Data were extracted onto standard data extraction forms independently by at least two reviewers (KKO and AMT and/or SAN). Data extracted included the study citation, study objectives, study design, length of study, time at which participants were assessed, inclusion and exclusion criteria for participants, characteristics of included participants (i.e., age, gender, stage of disease, comorbidity), description of intervention(s) (i.e., frequency, intensity, duration, type, level of supervision, location of intervention), types of outcome variables assessed and their values at baseline and study completion, and number of participants at baseline and study completion (including number of withdrawals). For the purposes of this review, constant exercise was defined as exercise at a constant intensity for a period of time. Interval exercise was defined as exercise conducted at a varied intensity for a total period of time. The reviewers met to achieve consensus regarding any difference in data interpretation or extraction from included studies that arose during the review process. In the case of missing data, authors were contacted in an attempt to obtain further information.

Two authors assessed the risk of bias in the included studies using the Cochrane Collaboration’s tool for assessing risk of bias [14]. Potential biases in studies may have included selection bias (random sequence generation and allocation concealment which may result in systematic differences in the comparison groups), performance bias (blinding of participants and personnel which could lead to systematic differences in the care provided apart from the intervention being evaluated), detection bias (blinding of outcome assessment that may result in systematic differences in outcome assessment), attrition bias (incomplete outcome data), and reporting bias (selective reporting of outcomes) [14].

We assessed the overall quality of evidence using the Grading of Recommendations Assessment, Development and Evaluation (GRADE) method [15]. We rated the quality of evidence for each outcome based on categories of very low, low, moderate and high [16]. We downgraded the evidence from high quality by one level for each of the following: attrition bias (where withdrawal rates were >15 %), performance bias (when participants were not blinded to the intervention), detection bias (when assessors of outcomes were not blinded to group allocation), publication bias (when publication bias was suspected), and inconsistency (when moderate I^2^ > 40 % or substantial I^2^ > 75 % heterogeneity exists) [17].

We produced a summary of findings (SoF) table for the main comparison of exercise versus no exercise with the following seven outcomes: immunological (CD4 count) and virological (viral load); cardiorespiratory (VO2max); strength (upper and lower body), weight (body weight); body composition (body mass index); and quality of life (SF-36 sub-scale scores). The SoF table was developed to illustrate the confidence in the effect estimates (quality of evidence) and magnitude of effect for seven key outcomes [18].

### Analysis

Outcomes were analysed as continuous and dichotomous outcomes whenever possible. Meta-analyses were performed using the random-effects model for outcomes using Review Manager (RevMan) computer software whenever there were sufficient data available in the studies, when similar or comparable outcome measures were used, and when participant comparison groups were similar [19].

For continuous outcomes, the weighted mean difference (WMD) and 95 % confidence intervals for the means were calculated whenever possible. For dichotomous outcomes, the odds ratio, absolute difference in odds, relative risk (RR), risk difference (RD), and the number needed to treat (NNT) and 95 % confidence intervals were calculated whenever possible. A p value of less than 0.05 indicated statistical significance for overall effect.

Subgroup analyses were performed whenever possible to estimate whether aerobic exercise interventions were associated with differences among groups using identified outcome measures.

We considered 50 cells/mm^3^ to indicate a clinically important change in CD4 count, 5 % to indicate a clinically important change in CD4 percentage, and 0.5 log10 copies to indicate a clinically important change in viral load. For cardiorespiratory outcomes, we considered 2 mL/kg/min to indicate a clinically important change in VO2max, 10 beats per minute to indicate a clinically important change in heart rate maximum (HRmax), and 5 min to indicate a clinically important change in exercise time. For strength outcomes we considered 5 kg to indicate a clinically important change in strength for lower extremities, 2 kg to indicate a clinically important change in strength for upper extremities. For weight and body composition outcomes, we considered 3 kg to indicate a clinically important change in body weight (which equals approximately 5 % of the average baseline body weight of participants), 5 cm to indicate a clinically important change in girth (waist and hip circumference), 3 cm to indicate a clinically important change in waist-to-hip ratio, 5 kg/cm^2^ to indicate a clinically important change in body mass index, 5 kg to indicate a clinically important change in fat mass, 5 % to indicate a clinically important change in percent body fat, and 5 cm^2^ to indicate a clinically important change in leg muscle area. For psychological outcomes, we considered 10 points to indicate a clinically important change in the sub scales of the SF-36 quality of life questionnaire; and 5.6 to indicate a clinically important change in the sub scales of the Profile of Mood States (POMS) scale [20]. While no established minimal clinically important difference (MCID) exists for the SF-36 questionnaire specifically with people living with HIV, we drew from other literature with clinically important differences for the SF-36 with other chronic conditions which demonstrates a small change is approximately 10 points [21, 22]. No other established MCID values exist for the other outcomes. Authors based the *a priori* estimates based on a combination of clinical experience and interpretations in the individual included studies.

We considered a p value of less than 0.1 as statistical significance for heterogeneity between studies [23]. We considered I^2^ ≤ 40 as low heterogeneity, I^2^ > 40–75 % moderate and I^2^ > 75 % substantial heterogeneity [17]. In instances of lack of statistical significance for an overall effect, confidence intervals were assessed for potential trends that may suggest movement towards an increase or decrease in overall effect. In instances of statistical significance for heterogeneity, we performed sensitivity analyses and explained potential reasons for heterogeneity.

## Results

**Fig. 1:**
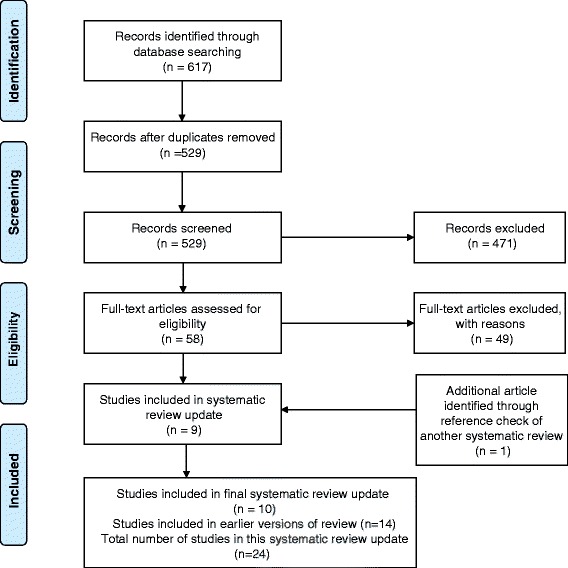
PRISMA Flow Diagram of Included Studies in Aerobic Exercise and HIV Systematic Review Update

Fourteen studies were included in the previous systematic review [10]. In this update, we retrieved a total of 529 citations, of which 58 were judged to merit scrutiny of the full article. Of the 58 studies reviewed, 11 met the inclusion criteria, one of which was a duplicate study [24] resulting in a total of 10 studies included in this fourth update [25–34] (Fig. 1-PRISMA Flow Diagram). Hence, a total of 24 studies (14 from the earlier review and 10 from the update) were included in this systematic review (See Table 1-Characteristics of Included Studies). An additional seven articles were identified as duplicate publications of studies included in the review: Kaushik [24] and Fitch [31] (in this update); and; LaPerriere [35] and LaPerriere [36]; Lox [37] and Lox [38]; Neidig [39] and Smith [40]; Fairfield [41] and Grinspoon [42]; Driscoll [43] and Driscoll [44]; and Mutimura [45] and Mutimura [46] (in the earlier review). In these instances, we extracted outcomes from all available sources but refer to the initial citation and/or the citation that included our primary outcomes of interest.

**Table 1:** Characteristics of Included Studies in the Aerobic Exercise and HIV Systematic Review (*n* = 24)

Study	Methods	Sample Size (at baseline)	% Women	% taking combination ART	Participants (at study completion)	Withdrawal Rate	Intervention	Duration and Frequency	Location of Exercise	Supervision
Agostini (2009)^a^ [34]	Randomized combined AER + PRE versus diet and aerobic exercise recommendation (no exercise) [2 groups] CONSTANT AEROBIC + PRE + DIET versus DIET and EXERCISE RECOMMENDATION ONLY	76	39 %	100 %	70	6/76 (8 %)	**EXERCISE (PRE + AER) + CONTROLLED DIET INTERVENTION GROUP**: Participants placed on a systematic and controlled diet and physical exerciser (aerobic activity of moderate intensity). *Aerobic*: walking on a treadmill for 40 min, run 30 min and stair climb for 15 min. *Anaerobic components included*: 40 min of PRE weight training in arms and legs; 10 cycles 3 repetitions. *PRE*: 2 kg for women and 5 kg for men. Cool Down and Relaxation: 5 min. Intensity: Medium intensity **DIET and AEROBIC EXERCISE RECOMMENDATION GROUP (CONTROL)**: Participants were given advice to follow a standard diet and physical exercise plan according to current recommendations.	70 min; 3X per week for 48 weeks	NR	NR
Baigis (2002) [50]	Randomized exercise and control groups [2 groups] CONSTANT AEROBIC versus NON-EXERCISING CONTROL	123	20 %	NR	69	54/123 (44 %)	**EXERCISE INTERVENTION GROUP**: Ski machine. 40 min total: 5 min stretching, 5 min warm-up on machine, 20 min constant aerobic exercise at 75–85 % HRmax followed by 5 min cool-down and 5 min stretching. **NON-EXERCISING CONTROL**: No detailed information.	40 min; 3X per week for 15 weeks	Home	Supervised
Balasubramanyam (2011)^a^ [33]	Randomized trial with five comparison groups 1) DIET+ EXERCISE (lifestyle change) plus 2 placebos versus 2) DIET + EXERICSE combined with niacin and fenofibrate versus 3) DIET + EXERCISE + niacin only plus 1 placebo versus 4) DIET + EXERCISE + fenofibrate only plus 1 placebo versus 5) USUAL CARE (with 2 placebos). [5 groups] ^*b*^ *Note for this systematic review we compared Group 1 (exercise + diet) to Group 5 (usual care).* CONSTANT AEROBIC + PRE + DIET versus DIET and EXERCISE RECOMMENDATION ONLY	191 (with dyslipidemia)	13 %	100 %	128	63/191 (33 %)	**DIET + EXERCISE INTERVENTION GROUP**: *Diet Intervention*: Participants were taught a weight-maintaining diet. *Exercise Intervention*: Participants engaged in an exercise program following ACSM guidelines. *Aerobic:* Participants began with 10 min stretching and 5 min warm-up; followed by 20–25 min of aerobic exercises (stationary bike and ergometer) at intensity of 70–85 % maximal heart rate or 60–80 % HR reserve, followed by 5–10 min cool down period. Intensity was measured using the modified Borg Rate of Perceived Exertion (RPE) scale. *PRE*: Resistive exercises were performed for 45–50 min; three sets of 8–12 repetitions with a rest break of 1–3 min between each set; followed by 5–10 min cool down. Intensity: 60–80 % 1 repetition maximum (1RM) of leg and bench press. After a given weight was lifted 8–12 times until muscular failure (unable to complete additional repetitions). Study trainers provided exercise plans to participants in this alternate program and reviewed their progress biweekly. **DIET AND EXERCISE RECOMMENDATION ONLY (USUAL CARE)**Participants received general advice on a heart healthy diet, kept a 7 day food record and received feedback on their caloric intake during a single baseline visit. Participants received a copy of “The Activity Pyramid” recommended by ACSM.	75-90 min; 3X per week for 24 weeks	Study gym	Supervised
Dolan (2006) [51]	Randomized exercise and control groups [2 groups] CONSTANT AEROBIC + PRE versus NON-EXERCISING CONTROL	40 (with self-reported and physical evidence of changes in fat distribution)	100 %	82 % taking ARVs (unclear whether it was cART)	38	2/40 (5 %)	**INTERVENTION GROUP** (Aerobic + PRE Exercise): Combined PRE and aerobic exercise for 2 h total. *Aerobic*: 5 min warm-up on stationary bike at 50 % estimated HRmax, followed by standard flexibility routine and aerobic and PRE exercise according to ACSM guidelines followed by a cool down period. *PRE*: concentric and eccentric phases of 6 selected upper and lower body muscle groups; Week 1: 3 sets of 10 reps for each muscle group at 60 % 1-RM, 3–5 s between reps rest, 2 min rest between sets, 4 min rest between muscle groups; week 3–16: 4 sets of 8 reps for each muscle group at 70 % 1-RM (Week 2–3), and 80 % 1-RM (week 4–16), 2–3 s between reps rest, 1 min rest between sets, 2 min rest between muscle groups. Each repetition lasted 6–10 s each. **NON-EXERCISING CONTROL GROUP**: Usual care	120 min; 3X per week for 16 weeks	Home	Supervised
Driscoll (2004a) [44]	Randomized combined exercise and metformin and metformin-only group [2 groups] CONSTANT AEROBIC + PRE + METFORMIN versus METFORMIN ONLY	37 (evidence of fat redistribution and hyperinsulinemia)	20 %	100 %	25	12/37 (32 %)	**INTERVENTION GROUP (Exercise + Metformin)**: Constant aerobic exercise followed by resistive training. Aerobic: 20 min aerobic exercise on stationary cycle at 60 % HRmax (week 1–2) and progressing to 30 min at 75 % HRmax (week 3–12) according to ACSM guidelines, 5 min warm-up on stationary bike, standard flexibility routine, followed by resistance training. *PRE*: of 3 sets of 10 repetitions for every muscle group, resting 2–3 s between repetitions, 2 min between sets, and 4 min between muscle group. Week 1: initial intensity of PRE was 60 % 1-RM; week 2–4 intensity increased to 70 % 1-RM; week 4–12 intensity of 80 % 1-RM. 1-RM was measured every other week and load adjusted to maintain relative intensity at 80 % 1-RM. **METFORMIN ONLY GROUP**: 500 mg of metformin twice per day, with a dose increase to 850 mg twice a day (week 2–12).	Total exercise time unknown (20-30 min aerobic;plus unknown duration of(PRE); Additional minutes (PRE); 3X per week for 12 weeks.	Hospital	Supervised
Farinatti (2010)^a^ [32]	Randomized exercise and control groups [2 groups] CONSTANT AEROBIC + PRE versus NON-EXERCISING CONTROL	27	NR	100 %	27	0/27 (0 %)	**INTERVENTION GROUP (Aerobic + PRE Exercise)**: Each 90 min session included aerobic, resistance and flexibility exercises. *Constant Aerobic Exercise*: cyclo-ergometer for 30 min at moderate intensity. *PRE*: 3 sets of 12 reps of 5 exercises at 60–80 % 12-RM. 1st week - 3 sets of 21 repetitions at 60 % 12 repetition maximum (12 RM) for all exercises. remaining weeks, the workload was 80 % of 12-RM for the following exercises: leg press, bench press, knee extension, seated bilateral row, abdominal sit-ups with rest intervals of 2–3 min between sets and exercises. *Flexibility*: 10 min - 2 sets of 30 s at maximal range of motion of 8 exercises (involving all major joints). **NON-EXERCISING CONTROL GROUP**: No intervention.	90 min; 3X per week for 12 weeks	NR	Supervised
Fitch (2012)^a^ [31]	Randomized trial: 1) exercise (lifestyle modification - LSM) and placebo (EXERCISE ONLY) versus 2) exercise (lifestyle modification) + metformin (EXERCISE + METFORMIN) versus 3) no LSM and metformin only (METFORMIN ONLY) versus 4) CONTROL (no LSM and placebo) [4 groups] CONSTANT AEROBIC + PRE VERSUS NON-EXERCISING CONTROL; and CONSTANT AEROBIC + PRE + METFORMIN versus METFORMIN ONLY	50 (with metabolic syndrome)	24 %	100 %	36	14/50 (28 %)	**INTERVENTION GROUP - EXERCISE (LIFESTYLE MODIFICATION)**: Included exercise 3 times per week (supervised) with dietary counselling once per week. *Aerobic*: Warm up was 5 min of stationary bike at 50 % maximum heart rate (220 minus age). Aerobic training was performed using a stationary cycle - each participant exercised for 20 min at 60 % their maximal HR (220-age) for the first 2 weeks followed by 30 min at 75 % their maximal heart rate for the duration of the study. [20 min total] *PRE*: The aerobic training was followed by 30 min of PRE on equipment. Exercises included: leg press, chest press, knee extension, lateral pull down, knee flexion, and triceps dip. Participants performed 3 sets of 10 reps for each exercise, their effort was increased over 6 months from 60 to 80 % of their 1RM. For those unable to reach 80 % 1RM the resistance was increased as tolerated. [30 min total] *Dietary Counseling*: Investigators covered a core curriculum modelled after a diabetes prevention program. The initial core sessions were completed within the first 18 weeks with review and reinforcement for the remainder of the study. **EXERCISE (LSM) + METFORMIN GROUP**: Exercise (or LSM) as per above plus 500 mg of metformin twice a day with a dose increase to 850 mg twice a day after 3 months.	50 min total (20 min aerobic; 30 min PRE); 3X per week for 52 weeks	NR	Supervised
Grinspoon (2000) [42]	Randomized trial with 4 comparison groups: 1) PRE + AEROBIC versus 2) PRE + AEROBIC + Testosterone versus 3) Testosterone only versus 4) Control [4 groups] CONSTANT AEROBIC + PRE versus NON–EXERCISING CONTROL	54 (with AIDS–related wasting)	0 %	72 %	43	11/54 (20 %) [4/26 (15 %) from the 2 comparison groups of interest; groups 1 and 4]	**INTERVENTION EXERCISE GROUP**: Supervised progressive strength training and constant aerobic conditioning. *Aerobic*: 20 min aerobic exercise on stationary cycle at 60–70 % HRmax, 15 min cool-down followed by resistance training. *PRE*: performed isotonically on computerized equipment and included: leg extension, leg curl, leg press, latissimus doris pull-down, arm curl, and triceps extension. 1-RM weight was established at baseline. *Intensity*: Participants increased resistance as follows: weeks 1 and 2, 2 sets at 8 repetitions per set, 60 % 1-RM; weeks 3 to 6, 2 sets, 8 repetitions per set, 70 % 1-RM; weeks 7 to 12, 3 sets, 8 repetitions per set, 80 % 1-RM.	Total exercise time unknown (20 aerobic + 15 cool-down + unknown duration of PRE) 3x per week for 12 weeks	NR	Supervised
LaPerriere (1990) [36]	Randomized exercise and control groups [2 groups] INTERVAL AEROBIC versus NON-EXERCISING CONTROL	50 gay men (unknown % who were HIV positive)^c^	0 %	NR	17 HIV positive participants	NR	**INTERVENTION GROUP**: Stationary bike 45 min total @ 80 % HRmax for 3 min, then @ 60–79 % HRmax for 2 min. **NON-EXERCISING CONTROL GROUP**: Usual care	45 min; 3X per week for 5 weeks	NR	NR
Lindegaard (2008)^a^ [30]	Randomized trial of aerobic versus progressive resistive exercise [2 groups] INTERVAL AEROBIC versus PRE	20 (with dyslipidemia, lipodystrophy)	0 %	100 %	18	2/20 (10 %)	**AEROBIC EXERCISE GROUP**: Aerobic exercise consisted of 8 different programs with 35 min of interval training. 5 min warm-up. Intensity varied from 50–100 % VO2max. The first 8 weeks the mean intensity was targeted at 65 % VO2max and the last 8 weeks were targeted to 75 % of VO2max. **PRE (RESISTANCE) EXERCISE GROUP**: PRE consisted of 8 exercises (leg curl, pull down, seated leg press, chest press, seated rows, leg extension, abdominal crunch and back extension) in resistance training machines for 45–60 min. The # of repetitions and sets changed every week. and the resting interval was 60–120 s.	Aerobic Session (35 min); PRE Session (45–60 min); 3X per week for 16 weeks	Public Fitness Centre	Supervised
Lox (1995) [38]	Randomized to two exercise groups (PRE and aerobic) and one control group [3 groups] CONSTANT AEROBIC versus PRE versus NON-EXERCISING CONTROL^b^	22 (aerobic and control groups only)	0 %	100 % (taking some form of ARV therapy that may or may not have been in combination)	21	1/22 (4 %)	**INTERVENTION GROUP (AEROBIC)**: Stationary bike, 45 min total: 5 min warm-up (stretching), 24 min cycle ergometer at 50–60 % heart rate reserve (HRR), 15 min cool-down. **INTERVENTION GROUP (PRE)**: 45 min total. Isotonic resistance to major muscle groups in legs, arms and upper body. Resistance was initiated at 60 % of an individual’s 1-RM and increased by either 5 or 10 lb at a time after successful performing 3 sets of 10 reps at constant weight.	45 min total; 3X per week for 12 weeks	NR	Supervised
MacArthur (1993) [53]	Randomised to two exercise intervention groups [2 groups] INTERVAL AEROBIC (HIGH versus LOW INTENSITY)	25	4 %	100 % taking ARV therapy but unclear how many were on combination ART.	6 (analysis based on those compliant with exercise program’ only	19/25 (76 %)	**HIGH INTENSITY EXERCISE-INTERVENTION GROUP**: High intensity exercise: 24 min total @75–85 % V02max x 4 min x 6 intervals. **LOW INTENSITY EXERCISE INTERVENTION GROUP**: Low intensity exercise: 40 min total @50–60 % V02max x 10 min x 4 intervals. Exercise included walking, jogging, biking, rowing and stair-stepping.	3X per week for 24 weeks	NR	NR
Maharaj (2011)^a^ [29]	Randomized trial comparing exercise versus non-exercising control [2 groups] CONSTANT AEROBIC versus NON-EXERCISING CONTROL	52	35 %	100 %	36	16/52 (31 %)	**AEROBIC EXERCISE (INCLUDING HOME EXERCISE) GROUP**: Participants were informed that they were to perform 20 min of cycling on a cycle ergometer, followed by 20 min of walking on a treadmill. This was followed by a home program of exercises and participants were shown how to monitor their respiratory, heart, and blood pressure at home. Home protocol = 10 min each of brisk walking, squatting with hands on the hips and jogging on the spot three times per week (total of 30 min). Intensity of Aerobic Exercise: Minimal resistance for 2 sessions of 10 min of cycling and 5 min of rest. This was followed by 2 sessions of 10 min of treadmill walking on a motorized treadmill with 5 min of rest (Modified Bruce protocol was used). Moderate Intensity included 50–70 % of age-predicted maximum heart rate (220 age in years) with heart rate maximum set within 10 beats of this percentage predicted maximum. **NON-EXERCISING CONTROL GROUP**: Participants received 20 min of minimal heat therapy to their thigh muscles of each leg by means of a shortwave machine. Home Protocol = reading a magazine at home for 30 min, 3 times per week.	Total of 40 min exercise and 20 min rest (Centre protocol) and total of 30 min (Home protocol); 4X per week (3X at home; 1X at centre) for 12 weeks	Rehabilitation Centre (1X per week) and Home (3X per week)	Supervised 1X per week at rehabilitation centre. Home protocol not supervised.
Mutimura (2008a) [45]	Randomized exercise and control groups [2 groups] CONSTANT AEROBIC versus NON-EXERCISING CONTROL	100 (with moderate to severe body fat redistribution)	60 %	100 %	97	3/100 (3 %)	**INTERVENTION GROUP (Aerobic Exercise)**: Six month supervised exercise programme at a fitness club in Kigali, Rwanda. *Aerobic Exercise*: ‘proper warm up’, stretching, and 15 min of brisk walking, followed by 45–60 min of jogging, running, stair climbing, low-back and abdominal stabilization and strengthening exercises, followed by a 15 min cool down and stretching exercises. Intensity: Gradual progression to encourage participants to perform jogging and running with the goal of achieving at least 45 % maximum heart rate (Weeks 1–3), 60 % maximum heart rate (Weeks 3–8), and 75 % maximum heart rate (Weeks 8–24). **NON-EXERCISING CONTROL GROUP**: No intervention	3X per week, (90 min per session, alternating days) for 24 weeks	Fitness club	Supervised
Ogalha (2011)^a^ [28]	Randomized exercise and control group [2 groups] AEROBIC + PRE + NUTRITION COUNSELING versus NUTRITION COUNSELING ALONE (CONTROL)	70 (lipodystrophy in 54 % of participants)	46 %	100 %	63	7/70 (10 %)	**EXERCISE + NUTRITIONAL COUNSELING (INTERVENTION) GROUP**: Participants engaged in 1 h supervised gym class 3 times per week plus monthly dietary counseling by a nutrition specialist. Intensity of exercise was 75 % maximum heart rate. **NUTRITIONAL COUNSELING (MONTHLY) NON-EXERCISING CONTROL GROUP**: Monthly dietary counselling by a nutrition specialist. Counseling sessions included 50 min discussion on dietary needs and recommendations. Participants also received a 30 min orientation on the importance of regular physical activities and how to include them in their daily routine. They were stimulated to perform activities like running, biking or walking for 1 h at least 3 times per week.	3X per week for 24 weeks	Fitness centre	Supervised
Perez-Moreno (2007)^a^ [27]	Randomized exercise and control groups [2 groups] CONSTANT AEROBIC + PRE versus NON-EXERCISING CONTROL	27 (prison inmates living with Hepatitis C co-infection)	0 %	10 %	19	8/27 (30 %)	**EXERCISE (AEROBIC + PRE) INTERVENTION GROUP**: 3 weekly sessions of 90-min duration each. Each session started and ended with a 10-min warm-up and cool-down period, respectively, consisting of cycle ergometer pedalling at very light workloads and stretching exercises for all major muscle groups. The 70-min core portion of the training session was divided into resistance and aerobic training. *PRE*: Resistance training included 11 exercises engaging 11 major muscle groups. *Stretching exercises*: involved an exercise performed at the end of each set of resistance exercise. In month 1, participants performed two and one set of exercises for large and small muscle groups and all sets were performed at a resistance that allowed 12–15 repetitions. Then, the resistance used was individually adjusted to allow the completion of 8–10 repetitions for three sets of the large muscle group exercises and two sets of the small muscle group exercises. The resistance used for each exercise was increased by 5–10 % when the participant could perform the prescribed maximal repetitions per set. After an increase in resistance, the repetitions per set typically decreased to the low end of the prescribed repetition range (12 or 8 repetitions). Abdominal crunches and low back extensions were performed in two sets of 15–20 repetitions at the start of the program and in three sets of 20 repetitions at the end. *Aerobic Exercise:* At the beginning of the program, aerobic training consisted of pedalling on a cycle ergometer for 20 min at 70 % of the age-predicted maximum heart rate. The duration and intensity of the sessions were gradually increased during the 4-month period so that participants completed 45 min of continuous pedalling at 80 % of HRmax by the end of the training program. For participants in the poorest physical condition, it was sometimes necessary to divide the first sessions into shorter time intervals to complete the total 20-min target duration. **NON-EXERCISING CONTROL GROUP**: Participants followed their usual sedentary lifestyle (physical activity level < 2; walking for a total of 30–60 min three days per week) and performing no strenuous exercise such as running, cycling, swimming or resistance training.	135 min total (PRE+Aerobic plus warm up and cool-down); 3X per week for 16 weeks	Prison	Supervised
Perna (1999) [48]	Randomized exercise and control groups [2 groups] INTERVAL AEROBIC versus NON-EXERCISING CONTROL	43	36 %	No participants were taking protease inhibitors but may have been taking other forms of ARV therapy	28	15/43 (35 %)	**INTERVENTION GROUP**: Stationary bike 45 min total @ 70–80%HR max x 3 min then 2 min “off” (10 min stretch pre and post). **NON-EXERCISING CONTROL GROUP**: Usual care	45 min total; 3 x per week for 12 weeks	NR	Supervised
Rigsby (1992) [47]	Randomized exercise and control (counselling) groups [2 groups] CONSTANT AEROBIC + PRE versus NON-EXERCISING CONTROL	45 (37 HIV+)	0 %	NR	31 (24 HIV+)	13/37 (35 %)	**INTERVENTION GROUP**: Stationary bike 60 min total @60–80 % HRreserve x 20 min (2 min warm-up and 3 min cool down at low intensity.) Stretching x 10–15 min. Strengthening x 20–25 min. **NON-EXERCISING CONTROL GROUP**: Received 90–120 min of counselling 1–2 times per week for 12 weeks.	3X per week for 12 weeks	NR	Supervised
Smith (2001) [40]	Randomized exercise and control groups [2 groups] CONSTANT AEROBIC versus NON-EXERCISING CONTROL	60	13 %	23 %	49	11/60 (18 %)	**INTERVENTION GROUP**: Minimum of 30 min constant aerobic exercise at 60–80 % V02 max consisting of mandatory 20 min walking/jogging on treadmill and remaining time spent either on stationary bicycle, stair stepper or cross-country machine. **NON-EXERCISING CONTROL GROUP**: Usual care	3x per week for 12 weeks	Exercise facility at medical centre	Supervised
Stringer (1998) [49]	Randomized to two exercise intervention groups and one control group [3 groups] CONSTANT AEROBIC versus NON-EXERCISING CONTROL	34	11 %	94 %	26	8/34 (24 %)	**MODERATE INTENTSITY (INTERVENTION #1)**: stationary cycle ergometer @ 80 % LAT x 60 min. **HEAVY INTENSITY (INTERVENTION #2)**: stationary cycle ergometer @ 50 % of difference between Lactic Acid Threshold (LAT) and VO2 max x 30–40 min. **NON-EXERCISING CONTROL GROUP**: Usual care	3 x per week for 6 weeks	NR	NR
Terry (1999) [54]	Randomized to two exercise intervention groups [2 groups] CONSTANT AEROBIC (MODERATE versus HEAVY INTENSITY)	31	33 %	NR	21	10/31 (32 %)	**MODERATE INTENSITY (INTERVENTION #1)**: Moderate exercise: walking @55–60 % HRmax x 30 min (5 min @ target intensity, 1 min recovery.) (15 min stretch pre and post) **HIGH INTENSITY (INTERVENTION #2)**: High exercise: running @75–85 % HRmax x 30 min (5 min @ target intensity, 1 min recovery) (15 min stretch pre and post) Exercise included walking, running and stretching.	3 x per week for 12 weeks	NR	NR
Terry (2006) [52]	Randomized to two groups (aerobic exercise + low lipid diet versus low lipid diet only) [2 groups] CONSTANT AEROBIC + LOW LIPID DIET versus LOW LIPID DIET ONLY	42 (with hyperlipidemia)	33 %	100 %	30	12/42 (28 %)	**INTERVENTION GROUP (Exercise + Low Lipid Diet)**: Constant aerobic exercise consisting of running for 30 min at 70–85 % HRmax, with 15 min stretching exercises to warm-up and 15 min to cool-down (total of 1 h). **NON-EXERCISING CONTROL GROUP (Low Lipid DIet Only)**: 45 min soft stretching and relaxation routines, three times a week also supervised by one of the investigators, without significant elevation of HR.	3X per week for 12 weeks	NR	Supervised
Tiozzo (2011) [26]^a^	Randomized exercise and control groups [2 groups] CONSTANT AEROBIC + PRE versus NON-EXERCISING CONTROL	37	39 %	100 %	23	14/37 (38 %)	**EXERCISE (AEROBIC + PRE) INTERVENTION GROUP**: Moderate Intensity *Aerobic Exercise*: Week 1 and 2: These 2 weeks were a phase-in period allowing participants to acclimate gradually to the exercise protocol. This consisted of 3 endurance sessions, 5 min warm up and cool down periods and 10–15 min of aerobic exercise utilizing a stationary treadmill or bike ergometer at an intensity of 60 % maximal heart rate. *Progression of Aerobic Intensity:* After the initial 60 % of aerobic training intensity and 60 % of 1RM resistance training intensity during the phase in period, intensity was gradually increased to 65 % of HRmax and 65 % of 1RM in Step 1, to 70 % in Step 2 and to 75 % in Step 3. *PRE*: All endurance sessions were followed immediately by core consisting of 8 two to three sets of 15 to 20 repetitions, and one set of 12 repetitions for ten exercises performed on stacked weight machines. The initial level for the resistance exercises was set at 60 % of one repetition maximum (1RM). *Progression of PRE Intensity*: In addition, Step 1 consisted of high repetitions (12), followed by lower repetitions in Step 2 and Step 3 (10 and 8 repetitions, respectively). Furthermore, similar to the phase-in period, other phases also allocated the same amount of time to each component (aerobic versus resistance) of the exercise program. **NON-EXERCISING CONTROL GROUP**: Participants were asked not to participate in any form of exercise.	3X per week for 12 weeks	Wellness medical centre	Supervised
Yarasheski (2011)^a^ [25]	Randomized exercise + pioglitazone versus pioglitazone only [2 groups] CONSTANT AEROBIC + PRE + PIOGLITAZONE versus PIOGLITAZONE ONLY	44 (with insulin resistance, impaired glucose intolerance and central adiposity)	13 %	100 %	39	5/44 (11 %)	**EXERCISE (AEROBIC + PRE) PLUS PIOGLITAZONE GROUP**: *Aerobic Exercise*: Stationary cycling, treadmill walk/jogging, stair stepper climbing, or elliptical training device. Target HR range during aerobic exercise was 50–85 % HR reserve (moderate to high intensity). During exercise, HR and time at the target HR were monitored. Signaled an alarm if target HR was not maintained. HR and time data were stored to verify adherence and response to the exercise. Trainer progressively increased the exercise intensity as the participants adapted. *PRE*: 4 upper and 3 lower body exercises following the aerobic session. Baseline 1 repetition maximum was measured during the 1st 3–4 exercise sessions on each of the machines. Initially PRE consisted of 1–2 sets of each exercise while lifting a weight that caused muscle fatigue/failure after 8 repetitions. The trainer monitored the participant’s exercise response daily and when the participant comfortably lifted the weight for 12 reps on any exercise, the weight (intensity) was increased by an amount 10 % that caused the muscle group to fatigue/fail after 8 reps. This progressive 8–12 repetition cycle was repeated for each exercise over the 4 month period. **PIOGLITAZONE ONLY GROUP**: Participants consumed a standard weight diet that contained adequate amounts of energy and macronutrients.	90-120 min session; 3X per week for 16 weeks	Indoor exercise facility	Supervised

*AER* aerobic exercise, *PRE* progressive resistive exercise, *NR* not reported, *cART* combination antiretroviral therapy, ACSM American College of Sports Medicine, LAT lactic acid threshold

^a^study included in this recent update of the systematic review

^b^For the purpose of this review, only the aerobic and control groups were included in meta-analyses

^c^LaPerriere (1990) [36] participants were not included in overall calculation of total number of participants because it was unclear how many participants were HIV positive in the baseline sample

### Included studies

All 24 included studies were randomized controlled trials. Fifteen studies included a non-exercising control group [26, 27, 29, 31, 32, 36, 38, 40, 42, 46–51]. Eight studies included co-intervention groups, comparing exercise plus diet or nutritional counselling versus diet or nutritional counselling only (Terry [52] low lipid diet; Ogalha [28] nutritional counselling; Balasubramanyam [33] low lipid diet plus exercise recommendation; Agostini [34] standard diet); exercise plus metformin versus metformin only (Driscoll [44]; Fitch [31]) exercise plus testosterone enanthate versus testosterone only (Grinspoon [42]) and exercise plus pioglitazone versus pioglitazone only (Yarasheski [25]). One study included a non-exercising counselling group (exercise vs. counselling group) [47]; two studies included a progressive resistive exercise (PRE) group compared with an aerobic exercise group [30, 38]; and two studies had comparison groups that compared heavy with moderate exercise [53, 54]. In 18 studies exercise was described as supervised [25–28, 30–33, 38, 40, 42, 44, 46–48, 50–52] and in the remaining four studies, the level of supervision was not stated. Two of the studies involved a supervised home-based exercise intervention [50, 51] and one study involved a combination of exercise at a rehabilitation center (supervised once per week) and home-based exercise (un-supervised three times per week) [29]. Ten studies involved exercise at a supervised facility such as a hospital [25, 44], medical or rehabilitation center [29, 40], exercise facility, fitness or wellness centre [26, 28, 30, 46], gymnasium [33], or prison [27]; whereas the location of exercise was not specified in the remaining 12 studies.

### Characteristics of participants

A total of 1242 participants were included in the review (number of participants in included studies at baseline). Participants included adults living with HIV at various stages of HIV infection, with CD4 counts ranging from <100 cells/mm^3^ to greater than 1000 cells/mm^3^. Studies included both men and women, with women comprising approximately 22 % of the total number of participants at study completion. The mean age of the participants in the included studies ranged from 30 to 49 years (inclusion criteria ranged from 18 to 65 years of age).

Four studies (17 %) were published prior to introduction of combination antiretroviral therapy (prior to 1996) [36, 38, 47, 53] followed by six (25 %) between 1998 and 2002 [40, 42, 48–50, 54], six (25 %) between 2004 and 2008 [27, 30, 44, 46, 51, 52] and eight (33 %) between 2009 and 2013 [25, 26, 28, 29, 31–34]. The majority of participants in 14 studies were taking combination antiretroviral therapy including 72 % [42]; 82 % [51] and 100 % on highly active antiretroviral therapy [25, 26, 28–34, 44, 46, 52]. Smith [40] reported 23 % of participants were taking protease inhibitors and all other participants were on some form of antiretroviral therapy. Five studies included participants who were not on combination antiretroviral therapy; however, others reported including participants taking some form of antiretroviral medication [38, 48–50] (Table 1).

Eleven studies included participants living with additional health-related conditions (in addition to HIV). Nine studies included participants with forms of hyperlipidemia [52], dyslipidemia [30, 33], lipodystrophy [28, 30], changes in fat distribution [44, 46, 51], hyperinsulinemia [44], insulin resistance, glucose intolerance and central adiposity [25], and metabolic syndrome [31]. One study included participants with AIDS wasting [42]. One study included participants who were co-infected with Hepatitis C and on methadone maintenance who were prison inmates [27]. Other personal characteristics were reported inconsistently across studies (Table 1).

### Outcomes of included studies

All except two included studies (92 %) assessed immunological or virological outcomes or both, in the form of CD4 count or viral load [29, 34]. Twenty of the 24 included studies (83 %) assessed cardiorespiratory outcomes [26–28, 30–33, 36, 38, 40, 44, 46–54]. Eleven of the 24 included studies (46 %) assessed strength outcomes [26, 27, 30–32, 38, 42, 44, 47, 48, 51]. Eighteen of the 24 included studies (75 %) assessed weight and body composition outcomes [25–28, 30–34, 38, 40, 42, 44, 46, 48, 51, 52, 54]. Thirteen of the 24 included studies (54 %) assessed psychological outcomes in the form of anxiety and depression, health status, depression, mood and life satisfaction, and health-related quality of life [26–29, 36, 38, 40, 46, 48–50, 53, 54]. Safety in the form of monitoring adverse events was reported in nine of the 24 studies (38 %) [25, 27, 29, 31–33, 47, 48, 51] (Table 2).

**Table 2:** Outcomes and Author’s Conclusions of Individual Studies Included in the Aerobic Exercise and HIV Systematic Review

Study	Immunological/Virological	Cardiorespiratory	Strength	Weight and Body Composition	Psychological	Adverse Events	Authors’ Conclusions
Agostini (2009)^a^ [34]	Not assessed	Not assessed	Not assessed	*Body Fat*: Decrease in abdominal fat was similar in both groups. There did not appear to be a significant difference between groups.	Not assessed	Not reported	Aerobic exercise and a balanced diet are key pillars in the non-pharmacological treatment of lipodystrophy.
Baigis (2002) [50]	*CD4 count*: No significant changes.	VO2max: No significant differences between exercisers versus non-exercisers. Results were attributed to the level of intensity and duration of exercise.	Not assessed	Not assessed	*Health-related quality of life*: Non-significant trend favouring exercisers compared to non-exercisers in HRQL. Significant improvement in overall health subscale of the MOS-HIV found among exercisers compared to non-exercisers.	Not reported	Exercise appeared to be safe in HIV-infected individuals.
Balasubramanyam (2011)^a^ [33]	*CD4 count and viral load*: No significant differences between groups.	No significant difference between groups for VCO2, VO2, respiratory quotient, resting energy expenditure.	Not assessed	*Weight*: No statistically significant difference between groups. *Body composition*: No significant difference between groups for body mass index (kg/m2), waist circumference, hip circumference, waist to hip ratio, body cell mass, fat mass (kg) and body fat (%). As intended in the Diet and Exercise (weight maintaining lifestyle intervention), there were no significant changes within groups or between groups in weight or BMI.	Not assessed	Adverse events were reported in both groups. Exercise Group: 24 adverse events reported in at least 1 % of participants ranging from (but not limited to) events such as diarrhea, nausea and vomiting, fatigue, dizziness, and headache. Recommendation Group: 20 adverse events reported in at least 1 % of participants ranging from (but not limited to) events such as: triglyceride >1000 mg/dl, elevated bilirubin, abdominal pain.	The combination of niacin and fenofibrate together with diet and exercise (D/E) is more effective than lifestyle change alone or drug monotherapy with lifestyle change in improving HIV associated dyslipidemia. Diet and Exercise intervention alone did not improve lipid levels or adiponectin or induce statistically significant changes in any of the secondary (body composition) outcomes.
Dolan (2006) [51]	*CD4 count and viral load*: No significant changes.	*6MWT*: Significant improvements in exercise time as measured by submaximal exercise time and 6MWT distance among exercisers compared with non-exercisers. *VO2max*: Significant improvements among exercisers compared with non-exercisers.	Significant improvements in upper and lower extremity strength (7 measures) among exercisers compared with non-exercisers.	*Weight*: No significant change between groups. *Body Composition:* Significant increase in total cross-sectional muscle area and muscle attenuation among exercisers compared with non-exercisers. Significant decrease in waist circumference among exercisers compared with non-exercisers. No significant difference between group for body mass index, abdominal visceral tissue area, subcutaneous adipose tissue area, and total fat.	Not assessed	Authors reported 1 participant who had an exacerbation of asthma, and 1 participant had chest pain but neither were related to exercise.	A 16 week supervised home based PRE and aerobic exercise program improves measures of strength, cardiorespiratory fitness, and body composition among women living with HIV.
Driscoll (2004a) [44]	*CD4 count and viral load*: No significant changes.	*Exercise Time*: Significant improvements in endurance time on cycle ergometer during submaximal stress test in the exercise and metformin group compared with the metformin only group.	Significant increases in upper and lower extremity strength (five of six indices) in the exercise and metformin group compared with the metformin only group.	*Weight*: No significant changes in either group. *Body Composition*: Significant increases in cross-sectional muscle area, and significant decreases in waist-to-hip ratio and abdominal fat area in the exercise and metformin group compared with the metformin only group. No significant changes in body mass index in either group.	Not assessed	None reported	Exercise training and metformin significantly improve cardiovascular outcomes more than metformin alone in persons living with HIV with fat redistribution and hyperinsulinemia. Exercise training (aerobic and PRE) is well-tolerated and improves muscle strength and size as well as aerobic fitness in persons living with HIV.
Farinatti (2010)^a^ [32]	*CD4 count*: No significant changes in Cd4 count or CD4 % within or between groups.	Significant improvements within exercisers and significantly greater improvements among exercisers compared with non-exercisers (slope and intercept for HR-workload).	Significant improvement within exercisers and significantly greater improvements in leg press (12-RM) and seated row (12-RM) among exercisers compared with non-exercisers.	*Weight*: Not assessed *Body Composition*: No significant difference within or between groups for body mass index (kg/mg) or body mass (kg).	Not assessed	No adverse events.	HIV infected patients treated with HAART improve their strength and aerobic fitness as a result of a supervised exercise program of aerobic, strength and flexibility exercises with no negative effect on immune function.
Fitch (2012)^a^ [31]	*CD4 count and viral load*: No significant differences between groups.	*VO2max and Endurance Time*: Improvements in exercisers compared with non exercisers. No significant effect of metformin on cardiopulmonary measures. Significantly greater improvement in VO2max among the combined Metformin + Exercise group versus the control group. (*p* = 0.05). Significantly greater improvement in exercise duration (min) among the exercising groups (EXERCISE only group) and (EXERCISE + METFORMIN group) versus control. Significantly greater increase in exercise duration among the EXERCISE only group versus METFORMIN only group. (*p* = 0.006).	Exercise was associated with improvements in all strength parameters (*p* < 0.01) compared with non-exercisers. Significantly greater improvement in triceps strength, knee flexor strength, lat pull down, knee extension strength, chest press, leg press, among the exercising groups (EXERCISE only group) and (EXERCISE + METFORMIN group) versus control. Significantly greater increase in triceps strength, knee flexor strength, lat pull down, knee extension strength, chest press, leg press, among the EXERCISE only group versus METFORMIN only group.	*Weight*: Not assessed *Body Composition*: Intramyocellular lipid (IMCL) improved in exercisers compared to non-exercisers. Visceral adipose tissue decreased in participants randomized to metformin only versus control, although this was not significant. Extremity fat did not change significantly in response to exercise or metformin. Significant between group difference between the exercise and control groups (*p* < 0.05) whereby the exercise group had greater reduction in tibialis anterior intramyocellular lipid (IMCL) compared with control. Significant difference between the exercise and metformin only group whereby the exercise group had a greater reduction in tibialis anterior IMCL compared with the metformin only group. Assuming that reduction in cellular lipid is a good outcome this suggests exercise had a beneficial effect beyond control and metformin only for reducing cellular lipid. No significant difference between groups for change in body mass index (kg/m2), visceral adipose tissue (cm2), subcutaneous adipose tissue (cm2), total extremity fat (kg), and waist circumference (cm).	Not assessed	Two participants in the EXERCISE group experienced muscle strains related to the resistance training necessitating modification of weights. There were no serious adverse events and the exercise program was well-tolerated.	Metformin participants demonstrated significantly less progression of coronary artery calcification (CAC) whereas the effect of exercise on CAC progression was not significant. Metformin had a significantly greater effect on CAC than exercise. Exercise participants showed significant improvement in HDL, and cardiorespiratory fitness compared to non-exercisers. Metformin prevents plaque progression in HIV infected individuals with metabolic syndrome. Exercise demonstrates improvements in cardiopulmonary fitness and strength.
Grinspoon (2000) [42]	*CD4 count and viral load*: No significant changes with exercise or testosterone therapy either alone or together as a co-intervention.	Not assessed	No significant change in strength (note strength was tested isometrically, which may underestimate change in strength).	*Weight:* No significant changes in either group. *Body Composition:* Participants in the exercise only group showed significant increases in lean body mass, arm muscle area, leg muscle area, HDL cholesterol and significant decreases in AST level compared to non-exercising control group. No significant changes in and fat mass in either the exercisers or non-exercising control group.	Not assessed	No deaths or adverse events.	Exercise has a significant effect on lean body mass and muscle area independent of testosterone. Muscle mass and strength may further increase in response to combined exercise and testosterone therapy. Exercise was associated with an increase in HDL cholesterol whereas testosterone decreased HDL cholesterol. Exercise significantly increases muscle mass and offers cardio protective effects by increasing the HDL cholesterol in men with AIDS wasting. Exercise may be a strategy to reverse muscle loss in this population.
LaPerriere (1990) [35, 36]	*CD4 count*: Exercisers showed increase in CD4 count. Non-exercising control group showed decrease in CD4 count.	*VO2max:* No change in V02 max in non-exercising controls. Improvements in fitness level averaged 10 % change in VO2 max in both seronegative and seropositive exercisers.	Not assessed	Not assessed	*Depression-Dejection Symptoms*: Seropositive non-exercising controls showed significantly larger increases in anxiety and depression than intervention groups as measured by the tension-anxiety subscale and depression-dejection subscale of the profile of mood state (POMS) scale.	Not reported	Aerobic exercise is a beneficial stress management intervention which may be a useful strategy for attenuating an acute stressor such as post-notification of HIV status.
Lindegaard (2008)^a^ [30]	Not reported	*VO2max:* Significant increase in VO2max by 14.4 % in the aerobic group with no difference in the PRE group. Greater improvement in VO2max in the AEROBIC group versus the PRE group.	Significant increase in strength by 30 % in the PRE group and by 7.8 % in the aerobic group. The increase was more pronounced after strength training than after aerobic training.	*Weight and Body Composition*: PRE group had significant decrease in body weight, increase in lean body mass, decreased total fat and limb fat mass whereas the AEROBIC group demonstrated no changes in these outcomes.	Not assessed	Not reported	Strength training and endurance training improved insulin mediated glucose uptake but only in the PRE group and not AEROBIC group and caused a reduction in total fat mass. In conclusion, both AEROBIC and PRE training increases insulin sensitivity in HIV-infected patients with lipodystrophy whereas only strength training reduces trunk fat mass. Authors suggest an appropriate exercise program should include PRE and AER training to reduce the risk of cardiovascular disease among people with lipodystrophy.
Lox (1995) [38]	*CD4 count and viral load*: No significant changes.	*VO2max:* Significant improvements among exercisers compared to non-exercisers with greater improvements in the aerobic compared to the PRE and non-exercising control groups. *Heart Rate:* Non-significant decrease in submaximum HR in the PRE group compared to a non-significant increase in the non-exercising control group.	Significant improvements in the PRE and aerobic exercise groups compared to the non-exercising control groups. Significantly greater improvements as measured by 1-RM in the PRE group compared to the aerobic and non-exercising control groups.	*Body Weight*: Significant increases in weight among PRE and aerobic exercise groups. *Body Composition*: No change among all 3 groups in average body mass index, fat mass, and body fat percentage. Significant increases in lean body mass and sum of chest, arm and thigh circumference among PRE and aerobic exercise groups.	Significant improvements in mood and life satisfaction in both the aerobic and PRE exercise groups compared to the non-exercising control group. Significantly higher life satisfaction in the aerobic group compared with the PRE group.	Not reported	Exercise results in improvements in body composition, strength, cardiopulmonary fitness, and mood and life satisfaction for people living with HIV.
MacArthur (1993) [53]	*CD4 count*: No significant changes.	The high intensity exercise group may have obtained a greater training effect than the low-intensity group (not significant). Significant increases in compliant exercisers (*n* = 6) for V02 max (24 %), minute ventilation (13 %), and oxygen pulse (24 %). At 12 weeks HR rate pressure product and RPE all decreased significantly in a group of 10 participants.	Not assessed	Not assessed	General health questionnaire scores improved for the 6 compliant participants.	No detrimental hematologic or immunologic effects were noted. One participant in the somewhat compliant group and 3 participants in the non-compliant group died prior to the end of 24 week study (deaths were not attributed to the intervention).	Exercise training is feasible and beneficial for moderate to severely immunocompromised HIV-infected individuals.
Maharaj (2011)^a^ [29]	Not assessed	Not assessed	Not assessed	Not assessed	*Quality of Life:* Physical and mental health component summary scores of the SF36 questionnaire improved significantly from baseline in the exercise group compared with the non-exercising control group. Authors reported that all SF36 domain scores improved significantly greater for the exercise group compared with the control group (general health, mental health, role physical, role emotional, pain, physical functioning, social functioning, and energy).	None of the participants showed any adverse effects on their clinical status of CD4 counts, viral load, or increase in opportunistic infections, heart, respiratory and blood pressure either during or after the exercises.	Results support the positive benefits of a rehabilitation program of moderate intensity and home program of exercises for patients on HAART. Results show a significant increase in all domains of quality of life with a possible achievement of an increase in the function and participation of ADLs.
Mutimura (2008a) [45]	*CD4 count*: No significant differences between groups.	Exercise group achieved a higher heart rate and rate of perceived exertion (RPE) at the end of the 20 m multi-stage shuttle run test (20mMST). *V02max*: Significant improvements among exercisers compared with non-exercisers as measured by the 20mMST.	Not assessed	*Weight:* Not assessed *Body Composition:* Significant decrease in body mass index (BMI), percent body fat mas (BFM), waist circumference, and waist-to-hip ratio among exercisers whereas these outcomes remained unchanged or increased among non-exercisers. Significant decrease in sum of skim folds, and percent body fat mass (%) and total body fat redistribution score (BFR) among exercisers compared with non-exercisers. Significant decrease in triceps, biceps, subscapular, suprailiac, and sum of skinfold thickness decreased more in the exercisers compared with non-exercisers. No change in hip circumference in either group.	*Quality of Life:* Significant improvements in quality of life (QOL) on the psychological, independence, social relationships, HIV+ HAART-specific domains of QOL, and overall QOL score as measured by the World Health Organization Quality of Life HIV Instrument (WHOQOL-BREF) for exercisers compared with non-exercisers. No difference between groups on the physical QOL domain score.	Not reported	Exercise training positively improves body composition, cardiorespiratory fitness and several components of QOL in HAART-treated HIV+ African participants with Body Fat Redistribution. Results imply that exercise training is a safe, inexpensive, practical and effective treatment for evolving metabolic and cardiovascular syndromes associated with HIV and HAART exposure in resource-limited settings such as Su-Saharan Africa.
Ogalha (2011)^a^ [28]	*CD4 count:* Significant improvement in CD4 count in both groups.	*VO2max:* ‘Marginally’ significant (*p* = 0.05) improvement in VO2max for exercisers only. Statistically significant improvement (reduction) in resting heart rate in the exercise group only (within group difference).	Not assessed	*Weight:* No significant within or between group differences for body weight. *Body Composition:* Statistically significant improvement in muscle mass, resting heart rate, body fat percent, hip circumference (decrease) among the exercisers (within group difference only). Statistically significant improvement in BMI, and hip circumference (decrease) among the control group (within group difference). No significant difference within or between groups for waist circumference or waist to hip ratio.	*Quality of Life:* All SF36 domain scores improved significantly similarly for all domains in both groups except for the pain domain (whereby the control group was the only group to show significant improvement). Improvements in QOL were significantly greater for the exercise group compared with the control group for general health, vitality, and mental health.	None reported	Regular exercise coupled with nutritional guidance in people living with HIV significantly improves quality of life. Main findings suggest that the intervention promoted significant modifications in increase in muscle mass and reduction in fasting glucose, BMI, body fat, and hip circumference.
Perez-Moreno (2007)^a^ [27]	*CD4 count:* Significant increase in CD4 count among exercisers (within group only).	Statistically significant improvement in peak workload (Watts) among exercisers whereas there was a significant decrease (worsening) in the control group. *HRmax:* Significant improvement in heart rate peak (bpm) among exercisers. A significant combined effect of group and time was found for peak-completed workload (W), HRpeak, and rate of HR decrease at 1-min post exercise compared to attained HRpeak among exercisers.	Significant improvement among exercisers for strength whereas no change among non-exercisers. Significant improvement in the upper and lower body dynamic strength endurance (6RM) among exercisers (bench press, knee extensor strength) compared with non-exercisers.	*Body Composition*: No significant changes within groups for body mass. Mean estimated muscle mass significantly increased in the exercise group (within group only) with no change in the control group.	*Quality of Life:* Statistically significant improvement in QOL as measured by the QOL Assessment with a Scale from Spain in the exercise group (*p* < 0.01) whereas no change occurred in the control group.	No major adverse effects and no major health problems were noted in the participants from both groups over the training period.	A combination of cardiorespiratory and resistance training produces significant gains in cardiorespiratory capacity and dynamic strength endurance of incarcerated men who are HIV-HepC co-infected and enrolled in a methadone maintenance program for the treatment of opioid dependency.
Perna (1999) [48]	*CD4 count:* Adherent exercisers (attending >50 % of exercise sessions) increased CD4 count by 13 % whereas non-adherent exercisers decreased CD4 count by 18 %. Control participants showed a decreasing trend of CD4 count by 10 %.	*VO2max and other Cardiopulmonary Outcomes:* Significant increase in V02 max (12 %), 02 pulse (13 %), maximum tidal volume (8 %), and minute ventilation (VE) (17 %) among adherence exercisers. No significant differences were found in non-adherent exercisers and non-exercising control groups.	Significant increase in leg power by 25 % adherent exercisers and no change in non-adherent exercisers or non-exercising controls.	*Weight:* Not assessed *Body Composition:* Significant increase in body mass index among adherent exercisers.	*Physician-Rated Health Status:* No significant differences were noted of physician-rated health status (note this outcome was not considered a true measure of psychological status because it was not completed by patient self-report).	One hospitalization was reported during the course of the study.	Aerobic exercise may significantly increase CD4 count among symptomatic HIV+ individuals.
Rigsby (1992) [47]	*CD4 count:* No significant changes.	*Aerobic Capacity:* Significant increases in aerobic capacity were shown in the exercise group with no change in non-exercising control group. *Heart Rate and Total Time to Voluntary Exhaustion:* Significant decreases in HR and increases in total time exercise to voluntary exhaustion	Significant increases in chest press and leg extension in the exercise group.	Not assessed	Not assessed	One death reported in the counselling group during the course of the study and one death one month after the study. Of the 4 participants who dropped out of the exercise group, one died immediately after the study conclusion.	HIV+ men can experience significant increases in neuromuscular strength and cardiorespiratory fitness when prescribed and monitored in accordance with ACSM guidelines for healthy adults. Increased fitness may occur without negative effects on immune status.
Smith (2001) [40]	*CD4 count and Viral Load:* No significant changes in CD4 cell count, CD4+ percentage, and viral load in either group.	*Fatigue*: Significant decrease in exercisers compared with non-exercisers as exercisers were able to stay on the treadmill 1 min longer compared to non-exercising control group (significant decrease in fatigue). *Rate of Perceived Exertion (RPE):* No significant effect on RPE or FEV1 in either group (dyspnea). Significant improvements in V02max in the experimental group (2.6 ml/kg per min) compared with the non-exercising control group (1.0 ml/kg per min).	Not assessed	*Body Weight*: Exercise group showed significant decreasing trends in weight. *Body Composition:* Significant decrease in waist-to-hip ratio among exercisers (note many participants were above ideal body weight prior to exercise; thus decreases in both weight and body composition were considered favourable outcomes). Exercise group showed significant decreasing trends in BMI, triceps, central and peripheral skin folds, abdominal girth and waist-to-hip ratio.	*Depression*: Significant improvements in Centre for Epidemiological Studies Depression Scale (CES-D), Profile of Mood State and Depression-dejection subscale of POMS scale, and non-significant trend to improvement in Beck Depression Inventory in the exercise group compared to non-exercising control group (reported in Neidig 2003).	No adverse events reported	Supervised aerobic exercise training safely decreases fatigue, weight, BMI, subcutaneous fat and central fat in HIV-infected individuals. [Neidig 2003]: Exercisers showed reductions in depressive symptoms.
Stringer (1998) [49]	*CD4 count and Viral Load:* No significant changes in all three groups.	*Aerobic Capacity:* An intensity-related aerobic training effect was seen (heavy > moderate) relative to the non-exercising control group. *VO2max and Work Rate Max and Lactic Acid Threshold:* Significant increases in V02 max and Work Rate max in the heavy exercise group. LAT increased significantly in both intervention groups.	Not assessed	Not assessed	*Quality of Life:* Significant improvements in both exercise groups on the QOL questionnaire relative to the non-exercising control group. No significant differences in QOL between the two intervention groups.	No adverse events reported	Exercise training resulted in a substantial improvement in aerobic function (heavy > moderate group) while immune indices were essentially unchanged. QOL markers improved significantly with exercise. Exercise training is safe and effective in this group and should be promoted for HIV+ individuals.
Terry (1999) [54]	*CD4 count*: No significant changes.	*HRMax:* Peak HR was unchanged for both groups; peak systolic BP increased significantly only in high intensity group.	Not assessed	*Body Composition*: No significant change in body mass, body fat percentage, and body density in either intensity exercise group.	*Depression:* No significant changes detected in depression scores of the Montgomery-Asberg Depression Scale.	Not reported	HIV+ individuals can increase fitness levels with aerobic exercise at a range of intensities. HIV+ individuals can obtain cardiorespiratory benefits of exercise similar to seronegative individuals. Moderate exercise was not associated with an improvement in immunologic markers. High intensity had no shown harmful effects. Short term aerobic exercise programs may be safely recommended to HIV+ individuals for improvement in functional capacity.
Terry (2006) [52]	*CD4 count and Viral Load:* No significant changes.	*VO2max:* Significant improvements in exercise capacity as measured by VO2max on the maximal treadmill test for the combined exercise and diet group and no change seen in the diet only group. *HRmax*: No significant changes in either group.	Not assessed	*Body Weight:* Significant decreases in weight in both groups. *Body Composition*: Significant decreases in body mass index, waist-to-hip ratio, and percentage of body fat in both groups. Significant increases in body density in both groups. No difference between groups.	Not assessed	No participants withdrew from the study due to infection or illness.	HIV positive adults with hyperlipidemia, when engage in 3 months of aerobic exercise and a low lipid diet do not experience significant changes in triglycerides, total cholesterol, or HDL cholesterol levels (not shown here) but they do improve functional exercise capacity.
Tiozzo (2011) [26]^a^	*CD4 count and Viral Load:* Significant decrease in CD4 count among non-exercisers (control group) whereas CD4 count remained the same in the exercise group. Exercisers had significantly greater CD4 count at study completion compared with non-exercisers. No significant changes in viral load in either group.	*VO2max:* Significant increase (improvement) in VO2max compared with non-exercisers. *HRmax*: No difference in heart rate or diastolic blood pressure within or between groups. Significant difference between groups at baseline for systolic blood pressure - the exercise group had lower systolic blood pressure at baseline but at study completion the control group had significantly lowered their systolic blood pressure.	Significant difference within exercisers who demonstrated an increase in 1RM chest and 1 RM legs whereas there was no change in the control group. Significantly greater improvement in 1RM chest among exercisers compared with control.	*Body Weight:* No significant changes. *Body Composition:* No significant changes in hip circumference or waist-to-hip ratio in either the exercise or control group. Significant reduction in waist circumference among exercisers whereas the non-exercisers waist circumference increased.	*Quality of Life:* Exercisers had significant improvements in SF36 physical function sub scale and mental health sub scale, compared with non-exercisers who demonstrated a significant worsening from baseline.	Not reported	A three month supervised, and moderate intensity cardiorespiratory and resistance exercise training program performed three times a week, can result in significant improvements in physical characteristics and physical fitness and QOL among people living with HIV.
Yarasheski (2011)^a^ [25]	*CD4 count and Viral Load:* No significant changes.	Not assessed	Not assessed	*Body Composition:* Significant increase in thigh muscle area among exercisers compared with non-exercisers (within and between group difference) and non-exercisers had a decrease in thigh muscle area. No other significant within or between group differences in other body composition outcomes: body mass index, fat mass, fat free mass, trunk fat mass, limb fat mass, visceral adipose tissue, abdominal adipose tissue, right and left thigh subcutaneous fat, total hip bone mineral density, lumbar spine bone mineral density, hip or lumbar z-score.	Not assessed	No serious adverse events or complications reported	Overall, combined exercise intervention for diabetes prevention that includes diet and exercise is more effective than medication interventions alone.

*HRQL* health-related quality of life, QOL quality of life, *MOS-HIV* Medical Outcomes Study HIV Scale, *VO2max* maximum oxygen consumption, *6MWT* 6 min walk test, *BMI* body mass index

^a^study included in this recent update of the systematic review

### Correspondence with authors

We wrote to authors of 11 included studies for clarification and additional data, three of whom responded. Yarasheski provided additional data including mean change and standard deviations of body mass index outcomes and viral load outcomes [25]. Agostini clarified the intervention included a combination of aerobic and resistive exercise and provided more details on the intervention. Authors indicated they were not able to provide raw data on body weight, fat mass, muscle mass, or waist circumference (data were reported as % increase or decrease) [34]. We requested SF-36 Physical Component Scores (PCS) and Mental Component Scores (MCS) from Tiozzo who responded with data on the eight SF-36 sub-scale scores [26].

### Risk of bias

**Fig. 2:**
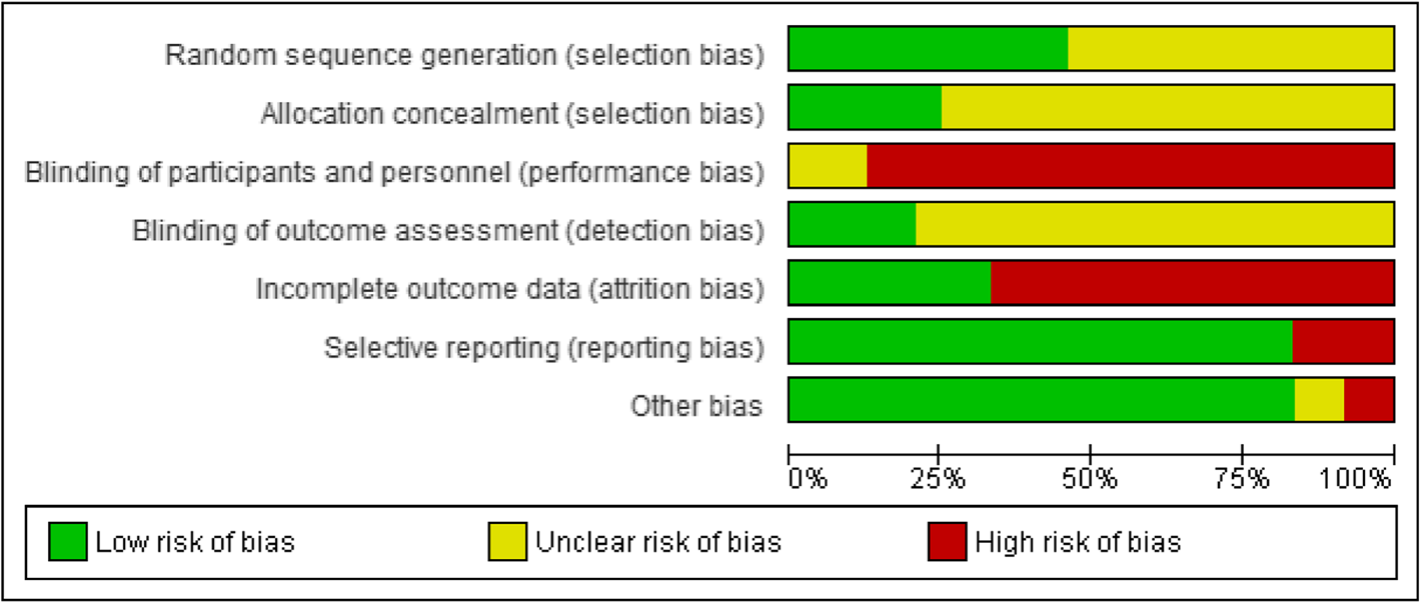
Cochrane Risk of Bias of Included Studies (*n* = 24 studies)

An overview of risk of bias of included studies is provided in Fig. 2. We describe details of potential bias of included studies below.

### Allocation (Selection Bias)

#### Random sequence generation

Authors of all 24 included studies reported that they used randomization to allocate participants to the comparison groups. However, an overall *unclear risk for selection bias* exists as 13 of the 24 included studies (54 %) did not include a description of the randomization process [25, 27, 28, 30, 32, 34, 36, 38, 40, 46–48, 54]. Low risk for selection bias was evident in the remaining eleven studies (46 %) that described the randomization process [26, 29, 31, 33, 42, 44, 49–53] (Fig. 2).

#### Allocation concealment

Overall an *unclear risk of selection bias* exists as 18 of the 24 included studies (75 %) did not describe the allocation sequence of participants. Six of the 24 included studies (25 %) had low risk for selection bias describing methods to conceal the allocation sequence of participants [27, 42, 44, 46, 50, 51] (Fig. 2).

### Blinding

#### Performance bias

Overall a *high risk of performance bias* exists across the included studies. Twenty-one of the 24 included studies (88 %) had a high risk for performance bias because participants were not blinded to the exercise intervention. Of these, five studies reported single-blinding of study personnel who were assessing outcomes to the group allocation [27, 29, 31–33]. In two studies, participants were blinded to co-interventions including metformin [31] and testosterone [42]. Nevertheless, we considered all of the above studies as high risk for bias given the inability to blind participants to the exercise intervention (Fig. 2). Unclear risk of performance bias occurred in three studies. In studies that compared aerobic to resistive exercise, different intensities of exercise, or where both comparison groups included some form of exercise, blinding was unclear [30, 34]. In MacArthur [53], participants were not told whether they had been randomized to the low-intensity group or the high-intensity group but it is unclear whether study personnel were blinded [53] (Fig. 2).

#### Detection bias

Overall an *unclear risk of detection bias* exists as 19 of the 24 included studies (79 %) did not provide sufficient information to determine whether study personnel were blinded to the outcome assessment. Five studies (21 %) had low risk for detection bias reporting blinding of study personnel who were assessing outcomes to group allocation [27, 29, 31–33] (Fig. 2).

### Incomplete outcome data (Attrition Bias)

Overall 303 participants withdrew from the included studies resulting in an overall ~24 % withdrawal rate (303/1242 participants at baseline). Withdrawal rates within individual studies ranged from 0 % [32, 38] to 76 % [53] (Table 1). Overall a high risk of attrition bias exists as 16 of the 24 included studies (67 %) reported withdrawal rates >15 %. The remaining eight included studies had low risk of attrition bias (33 %) with withdrawal rates less than 15 % [25, 28, 30, 32, 34, 38, 46, 51] (Fig. 2).

Withdrawal rates were similar between comparison groups for the majority of included studies. Three studies reported differences in the characteristics of participants who withdrew from the study [29, 33, 50] with more women and/or African Americans withdrawing from two studies [29, 50] and older participants with less familial history of diabetes remaining in the other study [33]. Twenty-three of the 24 included studies (96 %) made reference to participants who withdrew from or were non-adherent (or non-compliant) with the intervention. Withdrawal rates were not reported by LaPerriere [36]. Rates of withdrawal for individual studies are provided in Table 1.

Adherence to the exercise intervention was reported in nine of the 24 included studies (38 %) with adherence rates ranging from 61 % to 100 % [25–27, 30, 32, 46, 48, 51, 52]. Mutimura [45] reported an 82 % adherence rate and [48] reported 61 % of participants were adherent with the exercise intervention. Both studies defined adherence as attending >50 % of the exercise sessions [46, 48]. Farinatti [32] reported an 87 % adherence rate; Yarasheski [25] a 92 % adherence rate; and Perez-Moreno [27] an overall adherence rate to the intervention of 71 % [25, 27, 32]. Terry [52] reported that adherence to the exercise sessions was 100 % in both groups [52]. Dolan [51] reported that participants in the exercise group completed 96 % of the total exercise interventions. Tiozzo [26] reported that participants in the exercise group attended on average 81 % of the supervised exercise sessions [26]. Lindegaard [30] reported that adherence to exercise was 99 % and 96 % in the aerobic and resistive exercise groups, respectively. All nine of the studies that reported on adherence also supervised the exercise intervention [25–27, 30, 32, 46, 48, 51, 52]. MacArthur [53] reported six out of 25 of the participants were compliant with the exercise program (attended >80 % of the sessions), seven were somewhat compliant (attended 30–80 % of sessions) and 12 were not compliant (attended <30 % of sessions) [53]. Supervision of the exercise intervention was not reported in this study.

### Selective reporting (Reporting Bias)

Overall a *low risk of reporting bias* exists as the majority of included studies; 20/25 (83 %) were free of selective outcome reporting as authors provided data on all pre-specified outcomes. Four studies (17 %) had incomplete or inconsistent data [28, 33, 34, 53]. Agostini [34] provided outcome data for body weight, fat mass, muscle mass and waist circumference in % increase or decrease only. Authors responded to our request stating that they did not have access to the raw data. Balasubramanyam [33] did not report all outcomes for body composition and cardiorespiratory fitness. MacArthur [53] only reported on six of the participants who were compliant with the exercise intervention [53]. In Ogalha [28], the data across tables are inconsistent and authors did not report data for all outcomes such as viral load (Fig. 2).

### Other potential sources of bias

Overall a *low risk for other sources of potential bias* exists as the majority of studies (92 %) appeared free from other problems that could place a study at high risk of bias. Two studies possessed unclear risk of additional bias [32, 33]. Balasubramanyam [33] reported receiving > $10,000 funding from Abbott and research support from consultancy fees [33]. In Farinatti [32], a higher number of participants were assigned to the exercise group to maintain the sample size at study completion if adherence to exercise was low; it is unclear if this may have skewed results [32].

#### Group similarity at baseline

Sixteen of the 24 included studies (67 %) reported that comparison groups were similar at baseline [25, 27, 29–32, 34, 36, 40, 44, 46–50, 54]. MacArthur [53] and Grinspoon [42] did not report on group similarity at baseline [42, 53]. Lox [38] indicated significant differences between comparison groups for “most” participant characteristics, but variables that were different between the groups were not specified. Dolan [51] indicated that the exercise group had more endurance and knee strength at baseline. Terry [52] indicated that the low lipid diet group had a small but significantly higher haemoglobin level compared with the combined exercise and low lipid diet group [52]. Balasubramanyam [33] indicated that the family history of diabetes was less frequent in the diet and exercise intervention group compared with the non-exercising control group. Ogalha [28] indicated a small but significant difference in baseline fasting total cholesterol and high-density lipoprotein cholesterol closer to normality levels in the control group compared with the exercise group (mean rates were higher in the exercise group at baseline). Tiozzo [26] indicated that a higher number of participants in the exercise group had a smoking history compared with the control group placing them at higher risk of cardiovascular disorders. Faster mean heart rate recovery was evidence among participants in the control group compared with the exercise group at baseline [26].

### Meta-analyses - effects of interventions

Seventy-five meta-analyses were completed across eight sub-group comparisons in this review (17 of which included similar studies) resulting in 58 unique meta-analyses to this systematic review. Meta-analyses were performed for immunological and virological outcomes (CD4 count, CD4 percentage, and viral load), cardiorespiratory outcomes (VO2max, HRmax, exercise time), strength outcomes (chest press, leg press, knee extension, knee flexion, upper and lower body strength), weight and body composition outcomes (body weight, body mass index, lean body mass, fat mass, percent body fat, leg muscle area, waist circumference, hip circumference, waist-to-hip ratio), and psychological outcomes (quality of life, depression-dejection symptoms).

Of the 58 unique meta-analyses, 28 were new to this systematic review update, 16 were updated with additional studies, and 14 were the same as in the previous review [10]. Subgroup comparisons of the meta-analyses included 1) constant or interval aerobic exercise or combined aerobic and progressive resistive exercise (PRE) versus no exercise; 2) constant or interval aerobic exercise versus no exercise; 3) constant aerobic exercise versus no exercise; 4) interval aerobic exercise versus no exercise; 5) moderate-intensity aerobic exercise versus heavy-intensity aerobic exercise; 6) constant or interval aerobic exercise combined with PRE versus no exercise; 7) aerobic exercise versus PRE; and 8) combined aerobic exercise and diet and/or nutrition versus diet and/or nutrition only.

Fifteen of the 24 included studies compared constant or interval aerobic exercise or combined aerobic and progressive resistive exercise (PRE) with a non-exercising control group [26, 27, 29, 31, 32, 36, 38, 40, 42, 46–51]. Eight studies compared constant or interval aerobic exercise with a non-exercising control group [29, 36, 38, 40, 46, 48–50]. Seven studies compared combined aerobic and PRE [26, 27, 31, 32, 42, 47, 51]. Two studies compared aerobic with PRE [30, 38]. Three studies did not include a non-exercising control group [30, 53, 54] (Table 1). Driscoll [44] and Fitch [31] included aerobic and PRE combined with metformin compared with metformin only but we were unable to combine outcomes in meta-analysis. Terry [52], Ogalha [28], Balasubramanyam [33], and Agostini [34] included aerobic exercise or aerobic combined with PRE combined with a lipid diet and/or nutrition counselling versus diet and/or nutritional counselling alone. Yarasheski [25] included aerobic and PRE combined with pioglitazone versus pioglitazone only (Table 1).

The number of meta-analyses was limited due to variability in types of exercise interventions (aerobic exercise vs. combined aerobic and PRE exercise), level of exercise supervision, types of outcomes reported, and methodological quality. Aerobic interventions in the trials varied according to constant compared to interval exercise and moderate compared to heavy intensity exercise, and combined aerobic and resistive exercise compared to aerobic exercise alone. See Table 1 for characteristics of included studies and descriptions of specific interventions in individual studies.

### Heterogeneity

Heterogeneity (*p* < 0.1) was evident in 31 of the 58 unique meta-analyses (53 %). Reasons for heterogeneity may include differences in the types of participants in relation to antiretroviral use, body composition, comorbidity, gender, type and location of intervention, as well as methods of outcome measurement. We conducted sensitivity analyses on 18 of the 31 meta-analyses with heterogeneity (those with greater than two studies included in the meta-analysis. We discuss the specific sensitivity results and reasons for heterogeneity within the analyses below.

### Immunological and virological outcomes

Twenty-two of the 24 included studies (92 %) assessed immunological or virological outcomes, or both, in the form of CD4 count or viral load. Fourteen of the studies included a non-exercising control group, seven of which included combined interventions of aerobic and PRE [26, 27, 31, 32, 42, 47, 51]. Seven studies also measured immunological and virological outcomes but did not include a non-exercising control group [25, 28, 33, 44, 52–54].

#### CD4 count (cells/mm^3^)

Seven meta-analyses were performed for CD4 count. The majority (6 out of 7 meta-analyses) demonstrated no statistically significant changes in CD4 count between comparison groups (Table 3). Results demonstrated a non-significant trend towards an increase in change in CD4 count for participants in the aerobic or combined aerobic and PRE intervention group compared with the non-exercising control group; constant or PRE compared with no exercise; and significant increase in CD4 count for interval aerobic exercise compared with no exercise (Table 3). The point estimate in the latter two meta-analyses was above 50 cells/mm^3^, which suggests a trend towards a potential clinically important improvement in CD4 count among exercisers compared with non-exercisers.

**Table 3:** Results of Meta-Analyses in Aerobic Exercise and HIV Systematic Review: Immunological and Virological Outcomes

Outcomes	Sub-Group Comparison of Meta-Analysis	# of Individual Studies Included in Meta-Analysis	Number of Participants Included in Meta-Analysis	Weighted Mean Difference (WMD)	95 % Confidence Interval	*P* value of overall effect	I^2^ statistic (*p* value for heterogeneity)	Interpretation
CD4 count (cells/mm^3^)	Aerobic (constant or interval) exercise or combined aerobic and PRE compared with no exercise	14 studies (Baigis 2002 [50]; Dolan 2006 [51]; Farinatti 2010 [32]; Fitch 2012 [31]; Grinspoon 2000 [42]; LaPerriere 1990 [35, 36] ; Lox 1995 [38]; Mutimura 2008a [45]; Perez-Moreno 2007 [27]; Perna 1999 [48]; Rigsby 1992 [47]; Smith 2001 [40]; Stringer 1998 [49], Tiozzo 2011 [26])	479	37.43 cells/mm^3^	−0.16, 75.01	0.05	92 % (*p* < 0.00001)	No difference in change in CD4 count among exercisers compared with non-exercisers. Confidence interval indicates a positive trend towards an improvement in CD4 count among exercisers.
	Constant or PRE compared with no exercise	7 studies (Dolan 2006 [51]; Farinatti 2010 [32]; Fitch 2012 [31]; Grinspoon 2000 [42]; Perez-Moreno 2007 [27]; Rigsby 1992 [47], Tiozzo 2011 [26])	173	57.82 cells/mm^3b^	−1.27, 116.91	0.06	74 % (*p* = 0.0008)	No difference in change in CD4 count among exercisers compared with non-exercisers. Confidence interval indicates a positive trend towards an improvement in CD4 count among exercisers.
	Interval aerobic exercise compared with no exercise	2 studies (LaPerriere 1990 [35, 36]; Perna 1999) [48]	45	69.58 cells/mm^3b^	14.08, 125.09	0.01^a^	64 % (*p* = 0.09)	Significant increase in CD4 count for interval aerobic exercise compared with no exercise
	Constant or interval aerobic exercise compared with no exercise	7 studies (Baigis 2002 [50]; LaPerriere [35, 36] 1990; Lox 1995 [38]; Mutimura 2008a [45]; Perna 1999 [48]; Smith 2001 [40]; Stringer 1998 [49])	306	18.08 cells/mm^3^	−11.82, 47.99	0.24	82 % (*p* < 0.0001)	No difference in change in CD4 count among exercisers compared with non-exercisers.
	Constant aerobic exercise compared with no exercise	5 studies (Baigis 2002 [50]; Lox 1995 [38]; Mutimura 2008a [45]; Smith 2001 [40]; Stringer 1998 [49])	261	−3.11 cells/mm^3^	−31.06, 24.84	0.83	74 % (*p* = 0.004)	No difference in change in CD4 count among exercisers compared with non-exercisers.
	Combined aerobic exercise and diet and/or nutrition counselling group compared with diet and/or nutrition counselling alone	3 studies (Balasubramanyam 2011 [33]; Ogalha 2011 [28]; Terry 2006) [52]	161	−23.59 cells/mm^3^	66.10, 18.92	0.28	80 % (*p* = 0.006)	No difference in change in CD4 count among exercisers compared with non-exercisers.
	Moderate compared with heavy intensity exercise	2 studies (Stringer 1998 [49]; Terry 1999 [54])	39	−42.90 cells/mm^3^	−116.28, 30.47	0.25	44 % (*p* = 0.18)	No difference in change in CD4 count for participants exercising at moderate compared with heavy intensity.
CD4 Percentage (%)	Aerobic (constant or interval) exercise or combined aerobic and PRE compared with no exercise	3 studies (Baigis 2002 [50]; Farinatti 2010 [32]; Smith 2001 [40])	145	0.63 %	−1.32, 2.59	0.53	88 % (*p* = 0.0003)	No difference in change in CD4 percentage among exercisers compared with non-exercisers.
	Constant aerobic exercise group compared with no exercise *AND* Constant or interval aerobic exercise compared with no exercise	2 studies (Baigis 2002 [50]; Smith 2001 [40])	118	−0.33 %	−1.98, 1.32	0.69	76 % (*p* = 0.04)	No difference in change in CD4 percentage among exercisers compared with non-exercisers.
Viral Load (log10 copies)	Aerobic (constant or interval) exercise or combined aerobic and PRE compared with no exercise	6 studies (Dolan 2006 [51]; Fitch 2012 [31]; Grinspoon 2000 [42]; Smith 2001 [40]; Stringer 1998 [49], Tiozzo 2011 [26])	162	0.18 log10 copies	−0.13, 0.48	0.27	0 % (*p* = 0.68)	No difference in change in viral load among exercisers compared with non-exercisers.
	Aerobic exercise intervention group compared with no exercise *AND* Constant aerobic exercise group compared with no exercise	2 studies (Smith 2001 [40]; Stringer 1998 [49])	63	0.40 log10 copies	−0.28, 1.07	0.25	0 % (*p* = 0.88)	No difference in change in viral load among exercisers compared with non-exercisers.
	Combined aerobic and PRE group compared with no exercise	4 studies (Dolan 2006 [51]; Fitch 2012 [31]; Grinspoon 2000 [42], Tiozzo 2011 [26])	99	0.12 log10 copies	−0.23, 0.46	0.51	0 % (*p* = 0.46)	No difference in change in viral load among exercisers compared with non-exercisers.

^a^indicates statistical significance

^b^aligned with clinical importance

Meta-analyses resulted in no difference in change in CD4 count for constant or interval aerobic exercise compared with no exercise; constant aerobic exercise compared with no exercise; as well as combined aerobic exercise and diet and/or nutrition counselling group compared with diet and/or nutrition counselling alone (Table 3). Similarly, no difference in change in CD4 count was found for participants exercising at moderate compared with heavy intensity (Table 3).

#### Heterogeneity - CD4 Count

Six of the seven meta-analyses were statistically significant for heterogeneity (p < 0.1). Sensitivity analyses were conducted for five of the meta-analyses with greater than two studies. While removing combinations of studies reduced heterogeneity, sensitivity analyses did not change the overall effect of exercise on CD4 count beyond clinical importance.

#### CD4 percentage

Three meta-analyses were performed for CD4 percentage, two of which included similar studies. Meta-analyses demonstrated no difference in change in CD4 percentage for participants in the constant aerobic exercise group compared with the non-exercising control group; and no difference for participants in the aerobic or combined aerobic and PRE group compared with the non-exercising control group (Table 3).

#### Heterogeneity - CD4 percentage

One meta-analysis was statistically significant for heterogeneity (*P* < 0.1) (Table 3). Sensitivity analysis did not reduce heterogeneity, which was likely attributed to differences in characteristics of participants in the included studies.

#### Viral load (log10copies)

Four meta-analyses were performed for viral load, two of which included the same studies. Meta-analyses demonstrated no difference in change in viral load for participants in the aerobic exercise intervention group compared with the non-exercising control group as well as the constant aerobic exercise group compared with the non-exercising control group; no difference in the combined aerobic and PRE group compared with the non-exercising control group; and no difference for participants in the aerobic or combined aerobic and PRE intervention group compared with the non-exercising control group (Table 3). None of the meta-analyses were significant for heterogeneity.

#### GRADE rating - viral load

We are moderately confident in the non-significant effect estimate of 0.18 log10copies in viral load demonstrating no difference in change in viral load comparing aerobic exercise (or combined aerobic and PRE). The true effect is likely to be close to the estimate of effect, but there is a possibility that it may be substantially different. This outcome was downgraded from high to moderate GRADE quality of evidence due to incomplete outcome data (withdrawals of included studies were >15 %) (see Additional file 2 – GRADE Summary of Findings Table).

### Cardiorespiratory outcomes

Twenty of the 24 included studies (83 %) assessed cardiorespiratory outcomes, 13 of which compared aerobic or combined aerobic and PRE to non-exercise [26, 27, 31, 32, 36, 38, 40, 46–51]; seven of which compared constant or interval aerobic exercise to non-exercising control [36, 38, 40, 46, 48–50], three of which compared moderate aerobic exercise with heavy aerobic exercise [49, 53, 54], and six of which compared constant or aerobic exercise and PRE to non-exercising control [26, 27, 31, 32, 47, 51]. Driscoll [44] and Fitch [31] measured cardiorespiratory outcomes but compared exercise to metformin. Terry [52], Ogalha [28], and Balasubramanyam [33] measured cardiorespiratory outcomes but compared exercise combined with diet and/or nutritional counselling (Table 1).

#### VO2max

Six meta-analyses were performed for VO2max, five of which were significant favouring exercise compared with non-exercise. Meta-analyses showed a significant improvement in change of VO2max of 2.63 mL/kg/min for participants in the aerobic exercise intervention group compared with the non-exercising control group (Table 4); significant improvement in change of VO2max of 2.40 ml/kg/min for participants in the constant aerobic exercise group compared with the non-exercising control group (Table 4); significant improvement in VO2max of 3.71 ml/kg/min for participants in the combined aerobic and PRE group compared with the non-exercising control group (Table 4); significant improvement of 2.87 ml/kg/min for participants in the aerobic or combined aerobic and PRE group compared with non-exercising control group (Table 4); and a significant trend towards a greater improvement in VO2max of 4.30 mL/kg/min for participants in the heavy-intensity exercise group compared with the moderate-intensity exercise group (Table 4). No significant difference in change in VO2max was found for participants in the combined aerobic exercise and diet or nutrition counselling group compared with the diet or nutrition counselling group only (Table 4). All point estimates were greater than 2 mL/kg/min, which suggest a potential clinically important improvement in VO2max among exercisers and a greater improvement with heavy- versus moderate-intensity exercise.

**Table 4:** Results of Meta-Analyses in Aerobic Exercise and HIV Systematic Review: Cardiorespiratory Outcomes

Outcomes	Sub-Group Comparison of Meta-Analysis	# of Individual Studies Included in Meta-Analysis	Number of Participants Included in Meta-Analysis	Weighted Mean Difference (WMD)	95 % Confidence Interval	*P* value of overall effect	I^2^ statistic (*p* value for heterogeneity)	Interpretation
VO2max (ml/kg/min)	Aerobic (constant or interval) exercise or combined aerobic and PRE compared with no exercise	8 studies (Baigis 2002 [50]; Dolan 2006 [51]; Fitch 2012 [31]; Mutimura 2008a [45]; Perna 1999 [48]; Smith 2001 [40]; Stringer 1998 [49], Tiozzo 2011 [26])	358	2.87 ml/kg/min^b^	1.69, 4.04	<0.0001^a^	67 % (*p* = 0.003)	Significant (and potential clinically important) improvement in change in VO2max among exercisers compared with non-exercisers.
	Aerobic exercise (constant or interval) compared with no exercise	5 studies (Baigis 2002 [50]; Mutimura 2008a [45]; Perna 1999 [48]; Smith 2001 [40]; Stringer 1998 [49])	276	2.63 ml/kg/min^b^	1.19, 4.07	0.0003^a^	79 % (*p* = 0.0008)	Significant (and potential clinically important) improvement in change in VO2max among exercisers compared with non-exercisers.
	Constant aerobic exercise group compared with no exercise	4 studies (Baigis 2002 [50]; Mutimura 2008a [45]; Smith 2001 [40]; Stringer 1998 [49])	248	2.40 ml/kg/min^b^	0.82, 3.99	0.003^a^	83 % (*p* = 0.0006)	Significant (and potential clinically important) improvement in change in VO2max among exercisers compared with non-exercisers.
	Combined aerobic and PRE group compared with no exercise	3 studies (Dolan 2006 [51]; Fitch 2012; [31], Tiozzo 2011 [26])	82	3.71 ml/kg/min^b^	1.73, 5.70	0.0002^a^	0 % (*p* = 0.84)	Significant (and potential clinically important) improvement in change in VO2max among exercisers compared with non-exercisers.
	Heavy versus moderate intensity exercise	2 studies (MacArthur 1993 [53]; Stringer 1998 [49])	24	4.30 ml/kg/min^b^	0.61, 7.98	0.02^a^	67 % (*p* = 0.99)	Greater (and potential clinically important) improvement in VO2max for participants in the heavy-intensity exercise group compared with the moderate-intensity exercise group.
	Combined aerobic exercise and diet or nutrition counselling group compared with diet or nutrition counselling alone	2 studies (Ogalha 2011 [28]; Terry 2006 [52])	93	3.36 ml/kg/min^b^	−3.03, 9.75	0.30	88 % (*p* = 0.004)	No significant difference in change in VO2max was found for participants in the combined aerobic exercise and diet or nutrition counselling group compared with the diet or nutrition counselling group only
Maximum Heart Rate (bpm)	Aerobic (constant or interval) exercise or combined aerobic and PRE compared with no exercise	4 studies (Lox 1995 [38]; Perez-Moreno 2007 [27]; Perna 1999 [48]; Rigsby 1992 [47])	92	−7.33 beats per minute	−22.52, 7.87	0.34	97 % (*p* < 0.00001)	Non-significant trend towards a decrease in heart rate maximum among exercisers compared with non-exercisers.
	Aerobic (constant or interval) exercise or combined aerobic and PRE compared with no exercise	2 studies (Lox 1995 [38]; Perna 1999 [48])	49	−9.81 beats per minute	−26.28, 6.67	0.24	92 % (*p* = 0.0003)	Non-significant trend towards a decrease in heart rate maximum among exercisers compared with non-exercisers.
	Combined aerobic and PRE group compared with no exercise	2 studies (Perez-Moreno 2007 [27]; Rigsby 1992 [47])	43	−4.91 beats per minute	−34.13, 24.30	0.74	99 % (*p* < 0.00001)	No significant difference in change in heart rate maximum among exercisers compared with non-exercisers.
Exercise Time (min)	Aerobic (constant or interval) exercise or combined aerobic and PRE compared with no exercise	4 studies (Dolan 2006 [51]; Fitch 2012 [31]; Rigsby 1992 [47]; Smith 2001 [40])	129	2.66 min	0.12, 5.19	0.04^a^	98 % (*p* < 0.00001)	Significant increase in exercise time among exercisers compared with non-exercisers.
	Combined aerobic and PRE group compared with no exercise	3 studies (Dolan 2006 [51]; Fitch 2012 [31]; Rigsby 1992 [47])	83	3.29 min	0.10, 6.49	0.04^a^	97 % (*p* < 0.00001)	Significant increase in exercise time among exercisers compared with non-exercisers.

*bpm* beats per minute

^a^indicates statistical significance

^b^indicates potential clinically important improvement in outcome

#### Heterogeneity - VO2max

Four of six meta-analyses were statistically significant for heterogeneity (*p* < 0.1). Sensitivity analyses (three performed) indicated that removing Mutimura [45] from the aerobic versus non-exercise comparison, constant exercise versus non-exercise comparison, and aerobic or combined aerobic and PRE comparison successfully reduced heterogeneity. Results for overall effect remained statistically significant favouring exercise; however, the point estimate was reduced to 1.64 mL/kg/min (95 % CI: 1.06, 2.22), 1.53 mL/kg/min (95 % CI: 0.94, 2.12), and 1.99 mL/kg/min (95 % CI: 1.25, 2.73) respectively (not shown), which are below the threshold for clinical importance. Reasons for heterogeneity may be due to differences in characteristics of participants in the Mutimura [45] study; they were from Rwanda and all possessed moderate to severe body fat redistribution.

### GRADE rating - VO2max

We have very little confidence in the effect estimate demonstrating a significant increase of 2.87 ml/kg/min for VO2max comparing aerobic exercise (or combined aerobic and PRE) with non-exercising control. The true effect is likely to be substantially different from the estimate of effect (Table 4). This outcome was downgraded from high to very low on the GRADE quality of evidence due to incomplete outcome data (withdrawals of included studies >15 %), suspected publication bias, substantial heterogeneity (I^2^ = 67 %); and because the lower level of the confidence interval did not cross the estimated clinically important change in VO2max (despite the estimate surpassing our hypothesized clinically important change in VO2max of 2 ml/kg/min) (see Additional file 2 – GRADE Summary of Findings Table).

#### Maximum Heart Rate (HRmax)

Three meta-analyses were performed and showed a non-significant trend towards a decrease in HRmax of −9.81 beats/min, 7.33 beats/min and 4.91 beats/min for participants in the aerobic exercise intervention group compared with the non-exercising control group; aerobic or combined aerobic and PRE group compared with the non-exercising control; and combined aerobic and PRE compared with non-exercising control (Table 4), respectively.

#### Heterogeneity - maximum heart rate

Heterogeneity was present in all three meta-analyses (*p* < 0.1). Removing Perna [48] and Perez-Moreno [27] removed heterogeneity from the aerobic exercise or combined aerobic and PRE intervention versus non-exercise control comparison and the overall effect demonstrated a small but significant decrease in maximum heart rate of −19.21 beats per minute [95 % CI: −22.87, −15.55] (not shown). Reasons for heterogeneity may be due to differences in characteristics of participants in the included studies; participants in Perez-Moreno [27] were all in prison and co-infected with Hepatitis C.

#### Exercise time

Two meta-analyses were performed and significant increases in exercise time of 3.29 min were found for participants in the combined aerobic and PRE group compared with the non-exercising control group (Table 4); and 2.66 min for participants in the aerobic or combined aerobic and PRE group compared with the non-exercising control group (Table 4). Point estimates did not reach the 5 min threshold for clinical importance.

#### Heterogeneity - Exercise time

Both meta-analyses were statistically significant for heterogeneity (*p* < 0.1). Removing Rigsby [47] and Smith [40] from the aerobic exercise or combined aerobic and PRE intervention versus non-exercise control comparison and Rigsby [47] from the combined aerobic and PRE versus non-exercise control comparison removed heterogeneity and the overall effect remained significant, but was reduced to 1.72 min [95 % CI: 1.03, 2.42] among exercisers compared with control (not shown). Reasons for heterogeneity may be due to differences in characteristics of participants in the included studies.

See Table 2 for individual study results for outcomes unable to be combined in meta-analyses.

### Strength outcomes

Eleven of the 24 included studies (46 %) assessed strength outcomes [26, 27, 30–32, 38, 42, 44, 47, 48, 51]. Ten meta-analyses were performed, four of which included duplicate studies. Meta-analyses demonstrated significant improvements in upper and lower body strength as measured by increases in 1-repetition maximum for chest press, and knee flexion; and a non-significant improvement (trend) towards increases in 1-RM for leg press and knee extension for participants in the combined aerobic and PRE group versus non-exercising control group (Table 5). Two meta-analyses were conducted comparing aerobic versus resistive exercise. Significantly greater increases in strength were found among participants in the PRE group compared with participants in the aerobic exercise only group for upper and lower muscle groups (Table 5).

**Table 5:** Results of Meta-Analyses in Aerobic Exerciser and HIV Systematic Review: Strength Outcomes

Outcomes	Sub-Group Comparison of Meta-Analysis	# of Individual Studies Included in Meta-Analysis	Number of Participants Included in Meta-Analysis	Weighted Mean Difference (WMD)	95 % Confidence Interval	*P* value of overall effect	I^2^ statistic (*p* value for heterogeneity)	Interpretation
Chest Press (1-RM)	Combined aerobic and PRE group compared with no exercise	2 studies (Fitch 2012; [31], Tiozzo 2011 [26])	44	11.86 kg 1-RM^b^	2.37, 21.36	0.01^a^	46 % (*p* = 0.18)	Significant (and potential clinically important) improvement in change in chest press 1-repetition maximum among exercisers compared with non-exercisers.
Knee Flexion (1-RM)	Combined aerobic and PRE group compared with no exercise	3 studies (Dolan 2006 [51], Fitch 2012 [31]; Grinspoon 2000 [42])	81	10.46 kg 1-RM^b^	1.64, 19.29	0.02^a^	91 % (*p* < 0.00001)	Significant (and potential clinically important) improvement in change in knee flexion 1-repetition maximum among exercisers compared with non-exercisers
Leg Press (1-RM)	Combined aerobic and PRE group compared with no exercise	2 studies (Fitch 2012; [31], Tiozzo 2011 [26])	44	50.96 kg 1-RM^b^	−13.01, 114.92	0.12	88 % (*p* = 0.004)	Non-significant trend towards an increase in leg press 1-RM among exercisers compared with non-exercisers.
Knee Extension (1-RM)	Combined aerobic and PRE group compared with no exercise	3 studies (Dolan 2006 [51]; Fitch 2012 [31]; Grinspoon 2000 [42])	81	20.58 kg 1-RM^b^	−4.69, 45.86	0.11	95 % (*p* < 0.00001)	Non-significant trend towards an increase in knee extension 1-RM among exercisers compared with non-exercisers.
Upper Extremity Muscle Groups (1-RM)	Aerobic versus PRE	2 studies (Lindegaard 2008 [30]; Lox 1995 [38])	41	14.56 kg 1-RM or 3-RM^b^	10.63, 18.49	<0.00001^a^	32 % (*p* = 0.23)	Significantly (and potential clinically important) greater increase in strength among participants in the PRE group compared with the aerobic group.
Lower Extremity Muscle Groups (1-RM)	Aerobic versus PRE	2 studies (Lindegaard 2008 [30]; Lox 1995 [38])	41	23.09 kg 1-RM or 3-RM^b^	13.01, 33.18	<0.00001^a^	75 % (*p* = 0.04)	Significantly (and potential clinically important) greater increase in strength among participants in the PRE group compared with the aerobic group.

*1-RM* 1 repetition maximum, *3-RM* 3 repetitions maximum, *PRE* progressive resistive exercise

^a^indicates statistical significance

^b^indicates potential clinically important change in outcome

All six point estimates for upper and lower extremity strength are greater than 2 kg and 5 kg respectively indicating a clinically important greater increase in strength for resistive exercisers compared with aerobic exercise.

#### Heterogeneity - Strength

Heterogeneity was present in four meta-analyses. Removing Grinspoon [42] from the combined aerobic and PRE versus control comparison reduced heterogeneity (*p* = 0.95) for knee extension and the overall effect became significant for exercise compared with no exercise. Reasons for heterogeneity may be attributed to differences in study participants. Participants in Grinspoon [42] had signs of AIDS-related wasting.

### GRADE ratings - Strength

Our confidence is limited in the effect estimate of a significant increase of 11.86 kg for 1-repetition maximum for chest press comparing aerobic exercise (or combined aerobic and PRE) with non-exercising control. The true effect may be substantially different from the estimate of effect (Table 5). This outcome was downgraded from high to low on the GRADE quality of evidence due to incomplete outcome data (withdrawals of included studies were >15 %), publication bias suspected, and moderate heterogeneity (I^2^ = 46 %). However, the estimate demonstrated a significant effect for improvement in chest press and the lower limit of the confidence interval surpassed our hypothesized clinically important change in upper body strength (see Additional file 2 – GRADE Summary of Findings Table).

We have very little confidence in the effect estimate of a non-significant increase of 50.96 kg for 1-repetition maximum for leg press comparing aerobic exercise (or combined aerobic and PRE) with non-exercising control. The true effect is likely to be substantially different from the estimate of effect (Table 5). This outcome was downgraded from high to very low on the GRADE quality of evidence due to incomplete outcome data (withdrawals of included studies were >15 %), publication bias was suspected, and there was considerable heterogeneity (I^2^ = 88 %). Furthermore, the confidence intervals cross the clinically important improvement and deterioration for change in lower body strength (see Additional file 2 – GRADE Summary of Findings Table).

### Weight and body composition outcomes

Eighteen of the 24 included studies assessed weight and body composition outcomes [25–28, 30–34, 38, 40, 42, 44, 46, 48, 51, 52, 54].

#### Weight

Fourteen studies assessed body weight [25, 26, 28, 30, 33, 34, 38, 40, 42, 44, 46, 51, 52, 54]. Five meta-analyses were performed, two of which included the same studies. Meta-analyses demonstrated no difference in change in mean body weight for participants in the aerobic exercise group compared with the non-exercising control group as well as participants in the constant aerobic exercise group compared with the non-exercising control group; no difference in the combined aerobic and PRE group compared with the non-exercising control group; and no difference in the aerobic or combined aerobic and PRE group compared with non-exercising control (Table 6). Results also demonstrated no significant difference in change in body weight for participants in the combined aerobic and diet/nutrition counselling group compared with diet/nutritional counselling group only (Table 6).

**Table 6:** Results of Meta-Analyses in Aerobic Exercise and HIV Systematic Review: Weight and Body Composition Outcomes

Outcomes	Sub-Group Comparison of Meta-Analysis	# of Individual Studies Included in Meta-Analysis	Number of Participants Included in Meta-Analysis	Weighted Mean Difference (WMD)	95 % Confidence Interval	*P* value of overall effect	I^2^ statistic (*p* value for heterogeneity)	Interpretation
Mean Body Weight (kg)	Aerobic (constant or interval) exercise or combined aerobic and PRE compared with no exercise	5 studies (Dolan 2006 [51]; Grinspoon 2000 [42]; Lox 1995 [38]; Smith 2001 [40])	151	0.38 kg	−1.55, 2.31	0.70	48 % (*p* = 0.10)	No significant difference in change in body weight among exercisers compared with non-exercisers.
	Aerobic exercise (constant or interval) compared with no exercise *AND* Constant aerobic exercise compared with no exercise	2 studies (Lox 1995 [38]; Smith 2001 [40])	68	0.37 kg	−5.32, 6.05	0.90	71 % (*p* = 0.06)	No significant difference in change in body weight among exercisers compared with non-exercisers.
	Combined aerobic and PRE group compared with no exercise	3 studies (Dolan 2006 [51]; Grinspoon 2000 [42]; Tiozzo 2011 [26])	83	0.81 kg	−0.94, 2.56	0.37	19 % (*p* = 0.29)	No significant difference in change in body weight among exercisers compared with non-exercisers.
	Combined aerobic exercise and diet or nutrition counselling group compared with diet or nutrition counselling alone	3 studies (Balasumbramanyam 2011; Ogalha 2011 [28]; Terry 2006 [52])	161	−0.58 kg	−4.33, 3.17	0.76	93 % (*p* < 0.00001)	No significant difference in change in body weight for participants in the combined aerobic exercise and diet or nutrition counselling group compared with the diet or nutrition counselling group only.
Body Mass Index (kg/m^2^)	Aerobic (constant or interval) exercise or combined aerobic and PRE compared with no exercise	6 studies (Dolan 2006 [51]; Farinatti 2010 [32]; Fitch 2012 [31]; Lox 1995 [38]; Mutimura 2008a [45]; Tiozzo 2011 [26])	227	0.07 kg/m^2^	−0.52, 0.66	0.81	59 % (*p* = 0.03)	No significant difference in change in body mass index among exercisers compared with non-exercisers.
	Constant aerobic exercise compared with no exercise	2 studies (Lox 1995 [38]; Mutimura 2008a [45])	118	0.06 kg/m^2^	−1.89, 2.02	0.95	64 % (*p* = 0.10)	No significant difference in change in body mass index among exercisers compared with non-exercisers.
	Combined aerobic and PRE group compared with no exercise	4 studies (Dolan 2006 [51]; Farinatti 2010 [32]; Fitch 2012 [31]; Tiozzo 2011 [26])	109	0.21 kg/m^2^	−0.27, 0.68	0.40	0 % (*p* = 0.40)	No significant difference in change in body mass index among exercisers compared with non-exercisers.
	Combined aerobic exercise and diet or nutrition counselling group compared with diet or nutrition counselling alone	3 studies (Balasubramanyam 2011 [33]; Ogalha 2011 [28]; Terry 2006 [52])	161	−0.57 kg/m^2^	−1.26, 0.13	0.11	82 % (*p* = 0.004)	No significant difference in change in body mass index for participants in the combined aerobic exercise and diet or nutrition counselling group compared with the diet or nutrition counselling group only.
Lean Body Mass (kg)	Aerobic (constant or interval) exercise or combined aerobic and PRE compared with no exercise	4 studies (Farinatti 2010 [32]; Grinspoon 2000 [42]; Lox 1995 [38]; Perez-Moreno 2007 [27])	89	1.75 kg	0.13, 3.37	0.03^a^	16 % (*p* = 0.31)	Significant increase in lean body mass among exercisers compared with non-exercisers.
	Combined aerobic and PRE group compared with no exercise	3 studies (Farinatti 2010 [32], Grinspoon 2000 [42]; Perez-Moreno [27])	68	1.23 kg	−0.62, 3.08	0.19	17 % (*p* = 0.30)	No difference in lean body mass among exercisers compared with non-exercisers.
Leg Muscle Area (cm^2^)	Combined aerobic and PRE group compared with no exercise	2 studies (Dolan 2006 [51]; Grinspoon 2000 [42])	60	4.79 cm^2^	2.04, 7.54	0.0007^a^	11 % (*p* = 0.29)	Significant increase in leg muscle area among exercisers compared with non-exercisers.
Percent Body Fat (%)	Constant aerobic exercise compared with no exercise	2 studies (Lox 1995 [38]; Mutimura 2008a [45])	119	−1.12 %	−2.18, −0.07	0.04^a^	8 % (*p* = 0.30)	Significant decrease in percent body fat among exercisers compared with non-exercisers.
	Combined aerobic exercise and diet or nutrition counselling group compared with diet or nutrition counselling alone	2 studies (Ogalha 2011 [28]; Terry 2006 [52])	93	−2.35 %	−4.20, −0.50	0.01^a^	46 % (*p* = 0.17)	Significant decrease in percent body fat among participants in the combined aerobic exercise and diet or nutrition counselling group compared with the diet or nutrition counselling group only.
Fat Mass (kg)	Aerobic (constant or interval) exercise or combined aerobic and PRE compared with no exercise	4 studies (Dolan 2006 [51]; Fitch 2012 [31]; Grinspoon 2000 [42]; Lox 1995 [38])	102	0.15 kg	−0.59, 0.90	0.69	0 % (*p* = 0.82)	No difference in change in fat mass among exercisers compared with non-exercisers.
	Combined aerobic and PRE group compared with no exercise	3 studies (Dolan 2006 [51]; Fitch 2012 [31]; Grinspoon 2000 [42])	81	0.18 kg	−0.74, 1.10	0.70	0 % (*p* = 0.63)	No difference in change in fat mass among exercisers compared with non-exercisers.
Waist Circumference (cm)	Aerobic (constant or interval) exercise or combined aerobic and PRE compared with no exercise	5 studies (Dolan 2006 [51]; Fitch 2012 [31]; Mutimura 2008a [45], Smith 2001 [40])	224	−2.16 cm	−4.86, 0.54	0.12	82 % (*p* = 0.0002)	No difference in change in waist circumference among exercisers compared with non-exercisers.
	Constant aerobic exercise compared with no exercise	2 studies (Mutimura 2008a [45]; Smith 2001 [40]; Tiozzo 2011 [26])	142	−3.53 cm	−10.25, 3.19	0.30	94 % (*p* < 0.0001)	No difference in change in waist circumference among exercisers compared with non-exercisers.
	Combined aerobic and PRE group compared with no exercise	3 studies (Dolan 2006 [51]; Fitch 2012; [31])	82	−1.33 cm	−4.21, 1.54	0.36	37 % (*p* = 0.21)	No difference in change in waist circumference among exercisers compared with non-exercisers.
Hip Circumference (cm)	Aerobic (constant or interval) exercise or combined aerobic and PRE compared with no exercise	3 studies (Mutimura 2008a [45]; Smith 2001 [40])	165	−0.06 cm	−0.23, 0.11	0.50	0 % (*p* = 0.44)	No difference in change in hip circumference among exercisers compared with non-exercisers.
	Constant aerobic exercise compared with no exercise	2 studies (Mutimura 2008a [45]; Smith 2001 [40]; Tiozzo 2011 [26])	142	0.11 cm	−0.63, 0.85	0.77	35 % (*p* = 0.22)	No difference in change in hip circumference among exercisers compared with non-exercisers.
Waist-to-Hip Ratio (cm)	Combined aerobic and PRE group compared with no exercise	2 studies (Mutimura 2008a [45]; Smith 2001 [40])	142	−0.51 cm	−1.47, 0.45	0.30	100 % (*p* < 0.00001)	No difference in change in waist-to-hip ratio among exercisers compared with non-exercisers.
	Combined aerobic exercise and diet or nutrition counselling group compared with diet or nutrition counselling alone	2 studies (Ogalha 2011 [28]; Terry 2006 [52])	93	0.02 cm	0.01, 0.03	<0.00001^a^	0 % (*p* = 1.00)	Significantly greater increase in waist-to-hip ratio among participants in the combined aerobic exercise and diet or nutrition counselling group compared with the diet or nutrition counselling group only.

^a^indicates statistical significance

#### Heterogeneity - Weight

Heterogeneity was present in two of the five meta-analyses (*p* < 0.1). Removing Balasubramanyam [33] from the combined aerobic and diet/nutritional counselling versus non-exercise control comparison reduced heterogeneity (*p* = 0.24) but the overall effect remained non-significant (1.34 kg; 95 % CI: −0.24, 2.9) (not shown). Reasons for heterogeneity may be due to differences in the comorbidity of participants in the included studies. In Balasubramanyam [33], participants had dyslipidemia, in Ogalha [28], 54 % of participants had lipodystrophy and in Terry [52] participants had hyperlipidemia.

### GRADE rating - Weight

We are moderately confident in the effect estimate of a non-significant increase of 0.38 kg for body weight comparing aerobic exercise (or combined aerobic and PRE) with non-exercising control. The true effect is likely to be close to the estimate of effect; but there is a possibility that it is substantially different (Table 6). This outcome was downgraded from high to moderate on the GRADE quality of evidence due to incomplete outcome data (withdrawals of included studies were >15 %), and moderate heterogeneity (I^2^ = 48 %). The confidence interval limits and effect estimate do not cross our estimated clinically important change in body weight (see Additional file 2 – GRADE Summary of Findings Table).

#### Body composition

Eighteen studies assessed body composition [25–28, 30–34, 38, 40, 42, 44, 46, 48, 51, 52, 54]. Twenty-six meta-analyses were performed, each for body mass index, lean body mass, fat mass, percent body fat, leg muscle area, waist circumference, hip circumference, and waist-to-hip ratio. Eight of the 26 meta-analyses were duplicate and included the same studies.

#### Body mass index

Results demonstrated no difference in change in body mass index for four comparisons of participants in the aerobic or combined aerobic and PRE group compared with non-exercising control; constant aerobic exercise compared with non-exercising control; combined aerobic and PRE exercise group compared with non-exercising control and combined aerobic exercise and diet/nutrition counselling group compared with diet/nutritional counselling group only (Table 6).

### GRADE rating - Body Mass Index

We are very confident with the effect estimate of a non-significant increase of 0.07 kg/m^2^ for body mass index comparing aerobic exercise (or combined aerobic and PRE) with non-exercising control. The true effect is likely to be close to the estimate of effect; but there is a possibility that it is substantially different (Table 6). This outcome was not downgraded on the GRADE quality of evidence because this was an objective outcome of interest and publication bias was not suspected (see Additional file 2 – GRADE Summary of Findings Table).

#### Lean body mass

Meta-analyses demonstrated a significant increase in lean body mass of 1.75 kg for participants in the aerobic or combined aerobic and PRE group compared with participants in the non-exercising control (Table 6), No difference in lean body mass was found for participants in the combined aerobic and PRE exercise group compared with non-exercising control (Table 6).

#### Leg muscle area

Results demonstrated a significant increase in change in leg muscle area of 4.79 cm^2^ among participants in the combined aerobic and PRE group compared with the non-exercising control group (Table 6).

#### Percent body fat

Results also found a significant decrease in percent body fat of 1.12 % for participants in the constant aerobic exercise group compared with participants in the non-exercising control group and a significantly greater decrease in percent body fat of 2.35 % among participants in the combined aerobic exercise and diet or nutrition counselling group compared with diet or nutritional counselling group alone (Table 6).

#### Fat mass

Results demonstrated no difference in change in fat mass for two comparisons of participants in the aerobic or combined aerobic and PRE group compared with non-exercising control, and combined aerobic and PRE exercise group compared with non-exercising control (Table 6).

#### Waist and hip circumference and waist-to-hip ratio

No significant differences were found in change in waist circumference, hip circumference or waist-to-hip ratio for participants in the aerobic or combined aerobic and PRE group compared with non-exercising control; as well as participants in the constant aerobic versus exercise groups and combined aerobic and PRE exercise groups (Table 6). Results found a slightly greater increase in waist to hip ratio of 0.02 (95 % CI: 0.01 to 0.03) for participants in the combined exercise and diet or nutrition counselling group compared with diet or nutritional counselling group only however these results were not clinically important.

#### Heterogeneity - Body Composition

Heterogeneity was present in five meta-analyses for body mass index; waist circumference; and waist-to-hip ratio (*p* < 0.1). Removing Mutimura [45] from the aerobic or combined aerobic and PRE exercise intervention versus non-exercising control comparison reduced heterogeneity for body mass index (*p* = 0.41) and waist circumference (*p* = 0.16) but the overall effects remained non-significant (not shown). Removing Balasubramanyam [33] from the combined aerobic and diet/nutritional counselling versus non-exercise control comparison reduced heterogeneity for body mass index (*p* = 0.48) but the overall effect remained non-significant (not shown). Reasons for heterogeneity may be due to differences in participants in the included studies. The Mutimura [45] study was conducted in Rwanda opposed to the other included studies conducted in developed countries. In Balasubramanyam [33], participants had dyslipidemia, in Ogalha [28], 54 % of participants had lipodystrophy and in Terry [52] participants had hyperlipidemia.

### Psychological outcomes

Thirteen of the 24 included studies (54 %) assessed psychological outcomes in the form of anxiety and depression, health status, depression, mood and life satisfaction, and health-related quality of life [26–29, 36, 38, 40, 46, 48–50, 53, 54].

#### Health-related quality of life

Meta-analyses were performed for the eight sub-scales of the SF-36 questionnaire (2 studies; 59 participants). Results demonstrated statistically significant and clinically important improvements (>10 point change) on sub scales of mental health, role emotional and physical functioning sub scale scores, as well as statistically significant improvements in role physical, general health, and energy/vitality sub-scale scores of the SF36 questionnaire for participants in the aerobic or combined aerobic and PRE group compared with participants in the non-exercising control group. Results represent a clinically important improvement in mental health, role emotional and physical functioning compared to non-exercisers. A statistically significant decrease in pain sub-scale score was found indicating an increase in pain favouring non-exercisers compared with exercisers. No difference in change in social functioning was found between groups (Table 7).

**Table 7:** Results of Meta-Analyses in Aerobic Exercise and HIV Systematic Review: Psychological Outcomes

Outcomes	Sub-Group Comparison of Meta-Analysis	# of Individual Studies Included in Meta-Analysis	Number of Participants Included in Meta-Analysis	Domain	Weighted Mean Difference (WMD)	95 % Confidence Interval	*P* value of overall effect	I^2^ statistic (*p* value for heterogeneity)	Interpretation
Health-Related Quality of Life (SF36 Questionnaire)	Aerobic (constant or interval) exercise or combined aerobic and PRE compared with no exercise	2 studies (Maharaj 2011 [29])	59	General Health	4.73	1.72, 7.74	0.002^a^	0 % (*p* = 0.78)	Significant improvement in change in General Health subscale score favouring exercisers compared with non-exercisers.
			59	Mental Health	11.58^b^	1.35, 21.81	0.03^a^	87 % (*p* = 0.006)	Significant (and potential clinically important) improvement in change in Mental Health subscale score favouring exercisers compared with non-exercisers.
			59	Role Physical	6.56	3.17, 9.96	0.0002^a^	0 % (*p* = 0.53)	Significant improvement in change in Role Physical subscale score favouring exercisers compared with non-exercisers.
			59	Role Emotional	10.95^b^	8.19, 13.71	<0.0001^a^	0 % (*p* = 0.40)	Significant (and potential clinically important) improvement in change in Role Emotional subscale score favouring exercisers compared with non-exercisers.
			59	Pain	−6.59	−9.83, −3.36	<0.0001^a^	0 % (*p* = 0.40)	Significant reduction in change in Pain subscale score favouring non-exercisers compared with exercisers.
			59	Physical Functioning	16.30^b^	6.89, 25.72	0.0007^a^	67 % (*p* = 0.08)	Significant (and potential clinically important) improvement in change in physical function subscale score favouring exercisers compared with non-exercisers.
			59	Social Functioning	2.73	−4.84, 10.30	0.48	57 % (*p* = 0.13)	No difference in change in Social Functioning subscale score among exercisers compared with non-exercisers.
			59	Energy/Vitality	5.03	1.33, 8.72	0.008^a^	0 % (*p* = 71)	Significant improvement in change in Energy/Vitality subscale score favouring exercisers compared with non-exercisers.
			472	Overall Pooled Effect SF36 Subscale Scores	6.47	3.18, 9.75	<0.00001^a^	87 % (*p* < 0.00001)	Significant improvement in SF36 subscale scores favouring exercisers compared with non-exercisers.
Profile of Mood States (POMS) Scale	Aerobic (constant or interval) exercise compared with no exercise	2 studies (LaPerriere 1990 [35, 36]; Smith 2001 [40])	65	POMS Scale	−7.68^b^	−13.47, −1.90	0.009^a^	94 % (*p* < 0.0001)	Significant (and potential clinically important) improvement in depression-dejection scores favouring exercisers compared with non-exercisers.

^a^indicates statistical significance

^b^indicates potential clinically important improvement in outcome

#### Heterogeneity - Health-related quality of life

Two of the meta-analyses were significant for heterogeneity (*p* < 0.1) however we were unable to conduct sensitivity analyses given only two studies were included in the meta-analyses.

### GRADE rating - Health-related quality of life

We have very little confidence in the effect estimate demonstrating an improvement of 6.47 in SF-36 sub scale scores comparing aerobic exercise (or combined aerobic and PRE) with non-exercising control. The true effect is likely to be substantially different from the estimate of effect (Table 7). This outcome was downgraded from high to very low on the GRADE quality of evidence due to incomplete outcome data (withdrawals of included studies were >15 %), publication bias was suspected, and due to variable heterogeneity on specific meta-analyses of sub scales ranging from (I^2^ = 0 to 87 %). Nevertheless, the estimates for mental health, role emotional and physical function SF-36 sub scales surpass the estimated clinically important improvement in quality of life of 10 points; whereas the other 5 statistically significant sub scale analyses do not surpass the clinically important improvement estimate (see Additional file 2 – GRADE Summary of Findings Table).

#### Depression-dejection symptoms

One meta-analysis was performed and demonstrated a significant improvement in the depression-dejection sub scale of the Profile of Mood States Scale (POMS) by a reduction of 7.68 points for participants in the aerobic exercise intervention group compared with the non-exercising control group (Table 7). This represents a clinically important improvement in depression-dejection among exercisers compared to non-exercisers.

### Adverse events (Safety)

Safety in the form of monitoring adverse events was reported in 12 of the 24 studies (38 %) [25, 27, 29, 31–33, 40, 42, 47–49, 51] (Table 2). Meta-analysis was not possible due to the scarcity and variability of reporting adverse events. Adverse events were reported in five of the 24 studies, none of which were attributed to exercise or considered serious [31, 33, 47, 48, 51]. Rigsby [47] reported one death during the study in the counselling group; however, the death was not attributed to the exercise intervention. Perna [48] reported one hospitalization during the course of the study [48]. Dolan [51] reported that one participant in the exercise group had an exacerbation of asthma and one participant in the non-exercising group experienced chest pain at baseline . Balasubramanyam [33] indicated that adverse events (such as headaches, dizziness, flushing, diarrhea, nausea and vomiting and fatigue) were infrequent and events appeared to be similar across the comparison groups. Fitch [31] reported two participants in the exercise group experienced muscle strains related to the resistance training requiring modification of weights. Otherwise, no serious adverse events were reported and the exercise program was well tolerated.

Three studies reported no serious adverse events, health problems, or complications [25, 27, 32]. Two studies reported that no participants withdrew due to illness or infection [40, 49]. Grinspoon [42] reported having no reported deaths, adverse events or side effects during the course of the study. Maharaj [29] reported that none of the participants showed any adverse effects on their clinical status of CD4 counts, viral load, or increase in opportunistic infections, heart, respiratory and blood pressure either during or after the exercise intervention. The other studies did not report on any outcomes of adverse events (Table 2).

## Discussion

Ten additional studies were incorporated into this systematic review, eight of which were able to be included in meta-analyses [26–33]. This enabled us to perform 28 new meta-analyses for outcomes of CD4 count, VO2max, maximum heart rate, strength (chest press, leg press, knee extension and knee flexion, upper and lower muscle groups), weight, body mass index, percent body fat, lean body mass, waist circumference, hip circumference, waist-to-hip ratio, and quality of life. We were also able to incorporate additional studies into 16 meta-analyses from the previous review.

Meta-analyses suggest that performing constant or interval aerobic exercise, or a combination of constant aerobic and PRE for at least 20 min three times per week for at least five weeks appears to be safe and may lead to improvements in selected outcomes of cardiorespiratory fitness (maximum oxygen consumption, exercise time), body composition (lean body mass, leg muscle area, percent body fat), strength (chest press, knee flexors), and psychological status (quality of life, depression-dejection symptoms). We also found a trend towards potential clinically important improvements in cardiorespiratory fitness and psychological status. Results of this review suggest that aerobic exercise appears to be safe for adults living with HIV who are medically stable. This finding is based on the absence of reports of adverse events among exercisers within individual studies and the stability of CD4 count and viral load. Findings should be interpreted cautiously because results are based on participants who completed the exercise interventions and for whom there were adequate follow-up data.

Results of meta-analyses showed statistically significant improvements for outcomes of cardiorespiratory fitness (maximum oxygen consumption (VO2max); exercise time), strength (chest press, knee flexors), body composition (increase in lean body mass, leg muscle area, decrease in percent body fat), and psychological status (quality of life, depression-dejection symptoms).

Cardiorespiratory status results demonstrated potentially clinical important improvements in VO2max among aerobic exercisers compared with non-exercisers, combined aerobic and resistive exercisers compared to non-exercisers, and even greater improvements among participants doing heavy- versus moderate-intensity exercise.

This systematic review is the first to include meta-analyses for strength and quality of life, both of which demonstrated significant improvements among exercisers compared with non-exercisers. Strength results demonstrated potentially clinically important improvements in chest press and knee flexion, among combined progressive resistive and aerobic exercisers compared with non-exercisers. Greater clinically important improvements were found among resistive compared with aerobic exercisers suggesting a combination of aerobic and PRE may be ideal to maximize cardiovascular and strength benefits of exercise [55].

Weight and body composition results reached statistical significance but not clinical importance for lean body mass, leg muscle area, and percent body fat. Interpretations of changes in weight and body composition have shifted since the original review since the widespread use of combination antiretroviral therapy. Increases in body weight and lean body mass, and reductions in body fat may be interpreted as favorable outcomes as a reflection of increase in muscle mass and strength for adults living with HIV. Future updates of this review may be able to conduct sub-group analyses for studies conducted in the pre versus post-combination antiretroviral era.

Psychological status results demonstrated significant and clinically important improvements in quality of life and depression symptoms among exercisers compared to non-exercisers. Results demonstrated significant improvements for six of the eight SF-36 sub-scale scores, three of which demonstrated potential clinically important improvements for physical function, role emotional, and mental health. A significant decrease in pain sub-scale score on the SF-36 questionnaire for exercisers versus non-exercisers raised the query of whether exercisers may have experienced pain with exercise. This requires further study. Overall, with minimal adverse effects and few attributed to the interventions, exercise appears to be safe for adults living with HIV.

No significant differences were found in all but one meta-analysis for CD4 count or viral load outcomes, suggesting that aerobic exercise has little impact on immunological or virological status. These results were similar to those in previous versions of this review [10].

Results of this systematic review should be interpreted cautiously for a variety of reasons. Meta-analyses were limited due to variation in outcome measures used, comparison groups and types of interventions, enabling only two studies to be combined in the majority of meta-analyses [56]. Studies included in this review demonstrated a high risk of performance bias due to the inability to blind participants to the exercise intervention. This subsequently resulted in low GRADE ratings for quality of evidence (Additional file 2). The inability to blind participants to the aerobic exercise intervention may have resulted in a Hawthorne effect, whereby participants might perceive greater benefits associated with exercise based on the expectation that exercise should be linked to positive outcomes. In addition, the lack of assessor blinding may have resulted in assessor bias whereby assessors may have measured outcomes in favor of the exercise intervention. High risk of attrition bias was also evident due to incomplete data as many studies had withdrawal rates >15 %. Individual studies in this review included small sample sizes and high withdrawal or non-adherence rates (0–76 %). Participants who withdrew from the exercise program were often excluded from the results by the authors, resulting in a per protocol approach to the analysis. Thus, the overall findings among those who continued to exercise might not reflect the general experience of exercise among adults living with HIV. Nevertheless, authors often reported withdrawal rates were similar across comparison groups, and characteristics of participants who withdrew were similar to those who remained in the study, minimizing the potential for migration bias.

The majority of study participants were men between the ages of 18–65 years. This limits the external validity and ability to generalize results to women and older adults living with HIV and comorbidity. Furthermore, the maximum duration of aerobic exercise intervention was 52 weeks. Thus the long-term sustainable effects of aerobic exercise remain less clear. Next, despite combination antiretroviral therapy now increasingly reaching developing countries, this review only includes one study that assessed the impact of aerobic exercise in the developing country context [45, 46]. More recently, Ezema [57] similarly reported improvements in VO2max and CD4 count among aerobic exercisers compared with non-exercisers living with HIV in Nigeria. Recent evidence from South Africa also includes an investigation of the impact of home-based and pedometer walking interventions to improve physical activity among adults living with HIV [58, 59], demonstrating evidence on the role of exercise and physical activity for adults living with HIV in developing countries. Finally, recent evidence has also described the potential benefits of physical activity and exercise on mental health, neurocognitive outcomes, and activities of daily living for adults living with HIV [60–62].

Results of this systematic review are consistent with findings from previous iterations of this review concluding exercise is safe and beneficial for people living with HIV [10, 11]. Results are consistent with a systematic review that assessed the effect of combined twice weekly aerobic and resistive exercise on cardiorespiratory status, quality of life, physiologic and functional outcomes for adults living with HIV and similarly concluded that exercise is safe and beneficial for medically stable adults living with HIV [63, 64]. Gomes-Neto and colleagues concluded that aerobic exercise demonstrated benefits to cardiorespiratory status and quality of life, specifically improving body composition and aerobic capacity. Meta-analyses were not performed as part of this review; hence findings from our systematic review provide additional details on the pooled effect of aerobic exercise for adults living with HIV. Fillipas [65] conducted a systematic review and meta-analysis to investigate the effect of aerobic and resistive exercise on metabolic and body composition outcomes, including randomized controlled trials involving at least twice weekly exercise [65]. Results indicated that exercise resulted in decreased body mass index, triceps skinfold thickness, body fat percentage, waist circumference and waist-to-hip ratio [65]. We did not perform meta-analyses on metabolic outcomes as this was beyond the scope of this review; however the impact of exercise on metabolic health is increasingly important to consider in the era of combination antiretroviral therapy.

### Implications for research

Evidence about the safety and effectiveness of aerobic exercise for adults living with HIV is increasing, including narrative and systematic reviews, and clinical guidelines that support aerobic exercise interventions in the context of HIV [63–71]. Recent evidence-informed guidelines also highlight the potential benefits and role of aerobic and resistive exercise for older adults living with HIV [55, 72, 73].

Interpretations of weight and body composition outcomes should be considered relative to the publication dates of the included studies. Prior to the advent of combination antiretroviral therapy, studies on exercise tended to include participants with AIDS-wasting, whereas recent evidence includes participants on combination antiretroviral therapy with lipodystrophy, body fat redistribution, or hyperinsulinemia. These studies commonly assessed the impact of exercise on weight, body composition, and metabolic outcomes, and reflect our inclusion of weight and body composition in this review. Furthermore, an increasing number of studies include interventions with a combination of resistive and aerobic exercise, which led us to consider the effectiveness of combined PRE and aerobic exercise with non-exercise. We observed an increase in the number of studies that assessed interventions such as tai chi [74, 75] or co-interventions with exercise such as diet and/or nutritional counselling [28, 33, 34, 52] metformin [31, 44] and pioglitazone [25]. Finally, this review included only 22 % of women participants reflecting a largely under-represented population in the HIV and exercise literature.

Future studies should make efforts to include all participants in an “intent-to-treat” analysis, blind assessor of outcomes, and include clinically meaningful and standardized outcomes to strengthen existing meta-analyses. As the population living with HIV ages, future research should include older adults and those living with multiple concurrent health conditions such as cardiovascular disease, liver disease, kidney disease, and bone and joint disorders. As the HIV and exercise literature expands, future updates of this review should assess the impact of exercise co-interventions, the impact of exercise on outcomes including functional status and metabolic or inflammatory markers and make an attempt to conduct sub-group analyses to acknowledge the changing health-related consequences associated with the pre versus post-combination antiretroviral era. Finally, given the majority of studies (18/24) included exercise interventions in clinical settings supervised by health care or research personnel, future research may consider evaluating the effect of non-supervised exercise interventions or community-based fitness programmes that may reflect a self-management model of community-based exercise for people with HIV [76].

## Conclusions

Performing constant or interval aerobic exercise, or a combination of constant aerobic exercise and progressive resistive exercise three times per week for at least five weeks appears to be safe and can lead to significant improvements in outcomes of cardiorespiratory fitness (maximum oxygen consumption, exercise time), strength (chest press, knee flexors), body composition (lean body mass, percent body fat, leg muscle area), and psychological status (quality of life, depression-dejection symptoms). Greater increases in strength were found with resistive exercise compared with aerobic exercise. Interpretations of weight and body composition outcomes should be considered relative to the pre versus post combination antiretroviral therapy era. These findings are limited to participants who continued to exercise and for whom there were adequate follow-up data. Aerobic exercise is safe and beneficial for adults living with HIV who are medically stable.

## Availability of data and materials

Data supporting the findings can be found in the Tables. Data supporting the GRADE ratings can be found in Additional file 2. Additional data extracted from included studies may be shared upon request.

## Additional files

Additional file 1: Search Strategy Example for the Aerobic Exercise Systematic Review Update. (PDF 16 kb) Additional file 2: GRADE Summary of Finding Table: Aerobic (constant or interval) Exercise or Combined Aerobic and Progressive Resistive Exercise (PRE) Compared with No Exercise for adults living with HIV. (PDF 18 kb)

## Abbreviations

1-RM: 1 repetition maximum
BMI: body mass index
bpm: beats per minute
GRADE: Grading of Recommendations Assessment, Development and Evaluation
HIV: human immunodeficiency virus
PRE: progressive resistive exercise
