# Supplementary material for: Effectiveness of aerobic exercise for adults living with HIV: systematic review and meta-analysis using the Cochrane Collaboration protocol
Source: BMC Infect Dis. 2016 Apr 26;16:182. doi: 10.1186/s12879-016-1478-2 (PMC4845358; doi:10.1186/s12879-016-1478-2)
Supplement: Additional file 1: — Search Strategy Example for the Aerobic Exercise Systematic Review Update. (PDF 16 kb) [file 12879_2016_1478_MOESM1_ESM.pdf]

Additional File 1 – Search Strategy Example for the Aerobic Exercise Systematic Review Update

Database: Ovid MEDLINE(R) <1946 to March Week 4 2013>, Ovid MEDLINE(R) In-Process & Other Non-Indexed Citations <April 05, 2013>

Search Strategy:

----

1. exp HIV/ (78155)

2. exp HIV Infections/ (217346)

3. exp Anti-HIV Agents/ (48838)

4. Antiretroviral Therapy, Highly Active/ (15495)

5. exp HIV Long-Term Survivors/ (518)

6. (hiv or human immunodeficiency or acquired immunodeficiency syndrome or acquired immune deficiency syndrome or aids or hiv+).tw. (290585)

7. or/1-6 (328245)

8. limit 7 to aids (307148)

9. exercise/ or running/ or jogging/ or swimming/ or walking/ (98603)

10. Physical Exertion/ (51665)

11. Physical Fitness/ (20514)

12. exp Sports/ (103100)

13. "Physical Education and Training"/ (11396)

14. Exercise Therapy/ (23717)

15. exercise movement techniques/ or dance therapy/ or tai ji/ or yoga/ (2310)

16. Dancing/ (1718)

17. exercise.tw. (161755)

18. aerobic.tw. (50183)

19. or/9-18 (344409)

20. 8 and 19 (1266)

21. randomized controlled trial.pt. (346301)
22. controlled clinical trial.pt. (85685)
23. randomized.ab. (264221)
24. placebo.ab. (143103)
25. drug therapy.fs. (1598414)
26. randomly.ab. (192392)
27. trial.ab. (272836)
28. groups.ab. (1241713)
29. or/21-28 (3095863)
30. 20 and 29 (490)
31. exp animals/ not humans.sh. (3798097)
32. 30 not 31 (481)
33. limit 32 to yr="2009 -Current" (96)
34. remove duplicates from 33 (90)
35. limit 34 to "all child (0 to 18 years)" (18)
36. limit 35 to "all adult (19 plus years)" (13)
37. 35 not 36 (5)
38. 34 not 37 (85)

NOTE: search strategy was modified accordingly for the other databases.
