# Supplementary material for: Effectiveness of aerobic exercise for adults living with HIV: systematic review and meta-analysis using the Cochrane Collaboration protocol
Source: BMC Infect Dis. 2016 Apr 26;16:182. doi: 10.1186/s12879-016-1478-2 (PMC4845358; doi:10.1186/s12879-016-1478-2)
Supplement: Additional file 2: — GRADE Summary of Finding Table: Aerobic (constant or interval) Exercise or Combined Aerobic and Progressive Resistive Exercise (PRE) Compared with No Exercise for adults living with HIV. (PDF 18 kb) [file 12879_2016_1478_MOESM2_ESM.pdf]

Additional File 2 - GRADE Summary of Finding Table: Aerobic (constant or interval) Exercise or Combined Aerobic and Progressive Resistive Exercise (PRE) Compared with No Exercise for Adults Living with HIV

**Outcomes**	**Illustrative comparative risks (95% CI)**		**Number of Participants (studies)**	**Quality of the evidence (GRADE)**	**Comments**
	**No Exercise**	**Aerobic Exercise or Combined Aerobic and Progressive Resistive Exercise (PRE) Compared with No Exercise**			
**IMMUNOLOGICAL / VIROLOGICAL Viral Load (log10copies)**	The mean Viral Load (log10copies) in the control group was 0	The mean Viral Load (log10copies) in the intervention group was 0.18 higher (0.13 lower to 0.48 higher)	162 (6 RCTs)	⊕⊕⊕⊖ MODERATE	No significant difference in change in viral load between groups.
**CARDIORESPIRATORY VO2 Max (ml/kg/min)**	The mean VO2max (ml/kg/min) in the control group was 0	The mean VO2 max (ml/kg/min) in the intervention group was 2.87 higher (1.69 higher to 4.04 higher)	358 (8 RCTs)	⊕⊖⊖⊖ VERY LOW	***Significant improvement in VO2max among exercisers compared with non-exercisers.**
**STRENGTH Upper Body - Chest Press (1 repetition maximum; kg)**	The mean Chest Press (1 repetition maximum; kg) in the control group was 0	The mean Chest Press (1 repetition maximum; kg) in the intervention group was 11.86 higher (2.37 higher to 21.36 higher)	44 (2 RCTs)	⊕⊕⊖⊖ LOW	***Significant improvement in upper body strength among exercisers compared with non-exercisers.**

**STRENGTH**<br>**Lower Body - Leg Press (1 repetition maximum; kg)**	The mean Leg Press (1 repetition maximum; kg) in the control group was 0	The mean Leg Press (1 repetition maximum; kg) in the intervention group was 50.96 higher (13.01 lower to 114.92 higher)	44<br>(2 RCTs)	⊕⊝⊝⊝<br>VERY LOW	No significant difference between groups, but a trend towards an improvement in lower body strength among exercisers compared with non-exercisers.
**WEIGHT**<br>**Body Weight (kg)**	The mean Body Weight (kg) in the control group was 0	The mean Body Weight (kg) in the intervention group was 0.38 higher (1.55 lower to 2.31 higher)	151<br>(5 RCTs)	⊕⊕⊕⊝<br>MODERATE	No significant difference in change in body weight between groups.
**BODY COMPOSITION**<br>**Body Mass Index (kg/m2)**	The mean Body Mass Index (kg/m2) in the control group was 0	The mean Body Mass Index (kg/m2) in the intervention group was 0.07 higher (0.52 lower to 0.66 higher)	227<br>(6 RCTs)	⊕⊕⊕⊕<br>HIGH	No significant difference in change in body mass index between groups.
**QUALITY OF LIFE**<br>**SF-36 Subscale Scores**	The mean SF-36 Subscale Scores in the control group was 0	The mean SF-36 Subscale Scores in the intervention group was 6.47 higher (3.18 higher to 9.75 higher)	472<br>(2 RCTs)	⊕⊝⊝⊝<br>VERY LOW	***Significant improvement in quality of life scores among exercisers compared with non-exercisers.**

LEGEND: *Bold and asterisk text in the comments section indicates significant improvement for the outcome.
